# Iowa Implementation for Sustainability Framework

**DOI:** 10.1186/s13012-021-01157-5

**Published:** 2022-01-04

**Authors:** Laura Cullen, Kirsten Hanrahan, Stephanie W. Edmonds, Heather Schacht Reisinger, Michele Wagner

**Affiliations:** 1 Department of Nursing Services and Patient Care, University of Iowa Hospitals & Clinics, 200 Hawkins Dr., Iowa City, IA 52242 USA; 2 Department of Internal Medicine, University of Iowa, 200 Hawkins Dr., Iowa City, IA 52242 USA; 3 Institute for Clinical and Translational Science, University of Iowa, 200 Hawkins Dr., Iowa City, IA 52242 USA

**Keywords:** Implementation, Sustainability, Framework, Healthcare improvement, Strategies, Domain mapping

## Abstract

**Background:**

An application-oriented implementation framework designed for clinicians and based on the Diffusion of Innovations theory included 81 implementation strategies with suggested timing for use within four implementation phases. The purpose of this research was to evaluate and strengthen the framework for clinician use and propose its usefulness in implementation research.

**Methods:**

A multi-step, iterative approach guided framework revisions. Individuals requesting the use of the framework over the previous 7 years were sent an electronic questionnaire. Evaluation captured framework usability, generalizability, accuracy, and implementation phases for each strategy. Next, nurse leaders who use the framework pile sorted strategies for cultural domain analysis. Last, a panel of five EBP/implementation experts used these data and built consensus to strengthen the framework.

**Results:**

Participants (*n* = 127/1578; 8% response) were predominately nurses (94%), highly educated (94% Master’s or higher), and from across healthcare (52% hospital/system, 31% academia, and 7% community) in the USA (84%). Most (96%) reported at least some experience using the framework and 88% would use the framework again. A 4-point scale (1 = not/disagree to 4 = very/agree) was used. The framework was deemed useful (92%, rating 3–4), easy to use (72%), intuitive (67%), generalizable (100%), flexible and adaptive (100%), with accurate phases (96%), and accurate targets (100%). Participants (*n* = 51) identified implementation strategy timing within four phases (Cochran’s *Q*); 54 of 81 strategies (66.7%, *p* < 0.05) were significantly linked to a specific phase; of these, 30 (55.6%) matched the original framework. Next, nurse leaders (*n* = 23) completed a pile sorting activity. Anthropac software was used to analyze the data and visualize it as a domain map and hierarchical clusters with 10 domains. Lastly, experts used these data and implementation science to refine and specify each of the 75 strategies, identifying phase, domain, actors, and function. Strategy usability, timing, and groupings were used to refine the framework.

**Conclusion:**

The Iowa Implementation for Sustainability Framework offers a typology to guide implementation for evidence-based healthcare. This study specifies 75 implementation strategies within four phases and 10 domains and begins to validate the framework. Standard use of strategy names is foundational to compare and understand when implementation strategies are effective, in what dose, for which topics, by whom, and in what context.

**Supplementary Information:**

The online version contains supplementary material available at 10.1186/s13012-021-01157-5.

Contributions to the literature

• This study updated a widely used typology with 75 implementation strategies arranged within four phases and 10 domains, to promote effective implementation of evidence-based practices by clinician leaders.
• This study uniquely identified 10 domains that offer guidance for selecting implementation strategies and a bridge to the strategies’ potential mechanism of action.
• Implementation strategies included in the framework are unique to the implementation step within the EBP process, avoiding confusion among implementation, EBP process steps, and project management.

## Background

Adoption and sustained use of evidence-based practice (EBP) remains elusive [1–6]. The gap between research and practice is well known and a primary focus of implementation science. Adoption and sustainability begin when selecting, timing, operationalizing, and evaluating implementation strategies for use in practice. Clinicians are critical team members who must be brought into the EBP process early, before designing the practice change and implementation planning [7, 8].

Clinicians are challenged to select and use implementation strategies to improve clinical and operational outcomes. A large number of strategies for a variety of clinician users have been compiled [9–19]. Unfortunately, nurses and other clinicians continue to rely heavily on education and information sharing [20] as primary strategies for implementation [9–19], despite the lack of specificity about when or how to best use an informational approach [21].

**Fig. 1:**
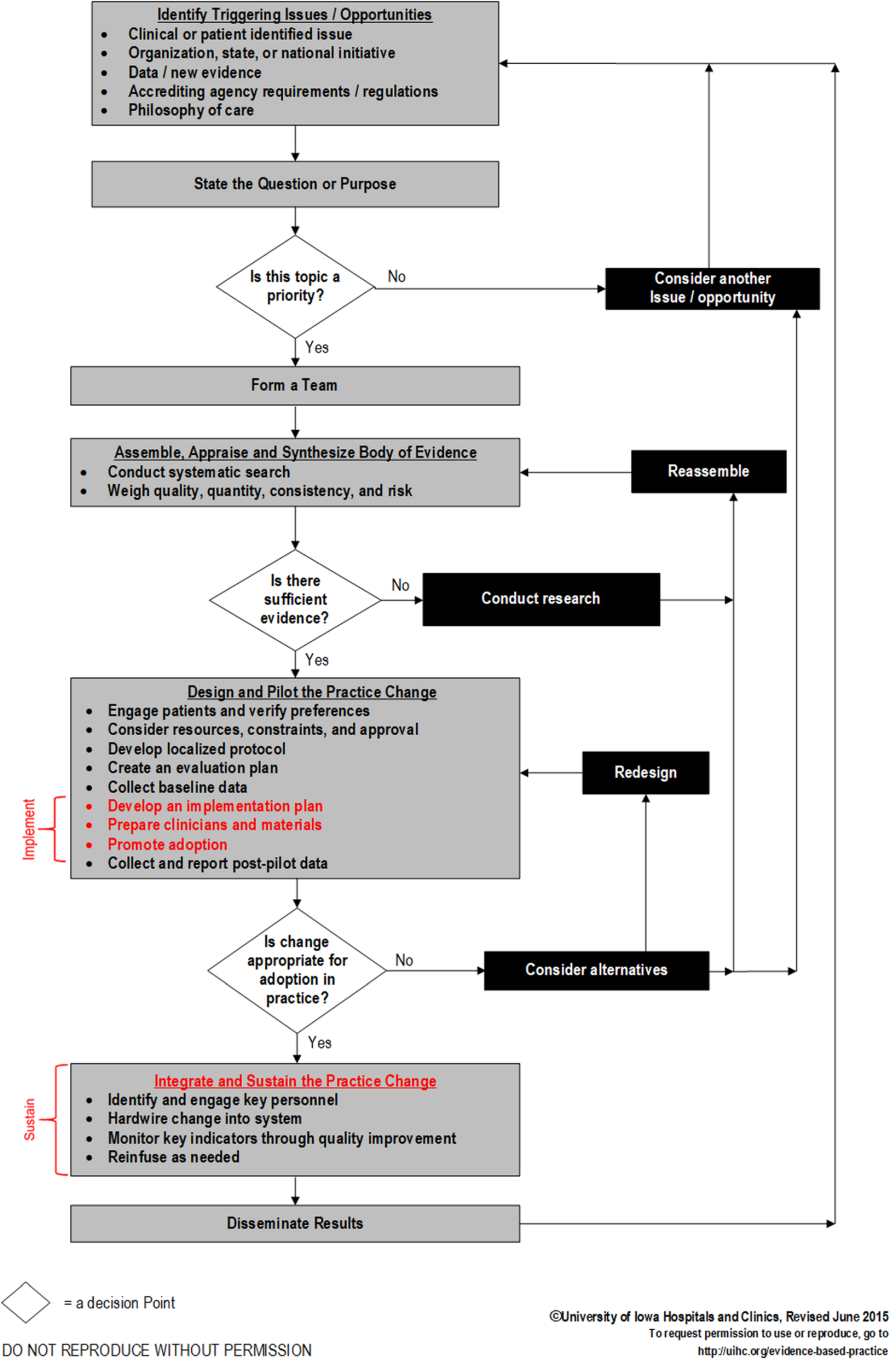
Iowa Model revised: Evidence-based practice to promote excellence in healthcare

Foundational to improving the science is the call to create a common language for implementation strategies [22–27]. Implementation strategies specifically target adoption, implementation, sustainability, and scale-up of EBP change [28]. In the Iowa Model (see Fig. 1), and other EBP process models, implementation strategies are explicitly differentiated from the EBP steps related to applying evidence to make decisions about care:

1. Identifying an issue or opportunity,
2. Stating the purpose,
3. Forming a team,
4. Assembling, appraising, and synthesizing the body of evidence,
5. Designing and piloting the practice change,
6. Integrating and sustaining the practice change, and
7. Dissemination.

Implementation strategies are also differentiated from project management processes, methods, skills, and knowledge which surround and support all EBP steps and are applied to achieve project goals (e.g., creating a charter, organizing, scheduling). To move implementation forward, scientists must take several additional steps [21, 25]: (1) confirm and reconcile these compilations of strategies, (2) define and specify strategies [29–31], (3) identify the link between the strategy and their mechanism of action [32], (4) describe how to bundle strategies, (5) operationalize strategies for use and testing [33], and (6) establish clarity in outcomes influenced by strategies [34–37], all while keeping implementation strategies actionable and feasible for use in clinical settings.

To this end, the need and recommendations for specifying elements of individual implementation strategies have been proposed [29–31, 36, 38]. Discrete strategies must be named, conceptually defined, and operationalized with further detail, so that each can be executed, measured, and compared in meaningful ways [31, 33]. Needed among these specifications is guidance matching local organizational needs with the strategy having the best fit to address that need [7, 33, 39, 40]. Further development and specification of an implementation framework to guide clinicians and researchers in strategy selection, while achieving sustainable outcomes, therefore, is needed.

**Fig. 2:**
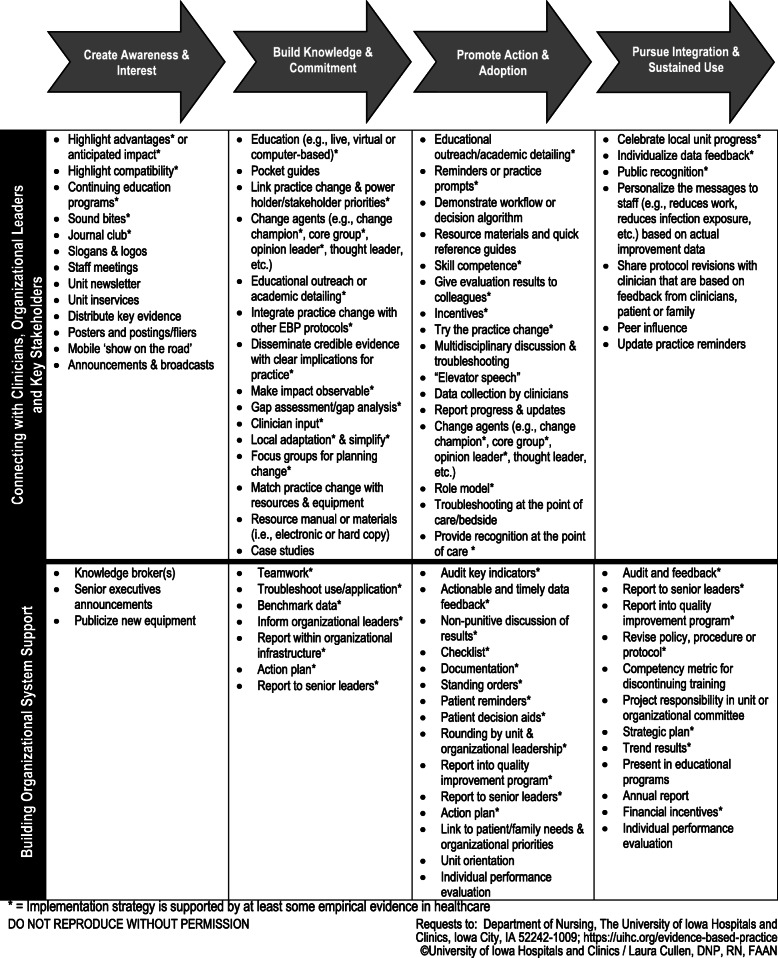
Implementation strategies for evidence-based practice

An application-oriented implementation framework, the *Implementation Strategies for EBP* guide (referred to as Iowa implementation framework in this paper; Fig. 2) [10], while designed as a framework for frontline nurses, is relevant for use by all clinicians and for research. The Iowa implementation framework was based on Diffusion of Innovations theory [40] to work synergistically within the Iowa Model, a well-known and frequently used EBP process model developed based on the same theory [8]. We have been leading EBP work since the 1980s and developed the implementation framework to fill a gap in the EBP process. Unlike other implementation strategy typologies, the strategies included in the Iowa implementation framework focus exclusively on the implementation step within the EBP process (see Fig. 1) and are arranged for clinicians as users to lead adoption among clinical teams. Users have requested this framework as a resource for implementing EBP within their healthcare settings, classroom teaching, and workshop presentations, and the most frequent request is for use in academic coursework. The framework includes a list of 81 implementation strategies with suggested timing for use within four implementation phases and targeting clinicians or the health system within which they work. The framework was developed to guide selection of strategies for leading EBP improvements and hardwire system changes. The strategies were identified and organized using implementation literature and decades of practical experience leading EBP improvements. Over the past 7 years, the guide has been requested from our organization’s website over 5000 times from 51 countries and all US states, cited over 100 times, and translated into several languages. The framework has been cited as supporting EBP change and organizational EBP programming, and as supporting the need for research or affirming the phased, yet iterative nature of implementation and the need to address organizational support [41–47]. Strategies have been operationalized with definitions, actionable procedures, and examples, to promote effective use and improve implementation outcomes in a monograph entitled *Evidence-Based Practice in Action* [33]. Despite the dissemination of these resources, the science has evolved and the need to promote evidence-based healthcare to improve quality and value continues to grow.

The purpose of this study was to evaluate and revise the Iowa implementation framework based on user feedback and emerging implementation science.

Specific aims were:

1. Determine user perspectives of usefulness of the framework
2. Evaluate the typology of implementation strategies
3. Evaluate the timing of implementation strategies within phases
4. Identify domains for related implementation strategies
5. Use study data, emerging science, and expert consensus to revise the framework

The goal was to evaluate and strengthen the framework to be feasible and actionable and improve the effective use of implementation strategies by clinicians and healthcare leaders responsible for promoting delivery of evidence-based healthcare, as well as promote its use among implementation researchers.

## Methods

A four-step, iterative mixed-methods approach guided framework evaluation and revisions: first—evaluate framework usability; second—identify and specify strategies; third—identify strategy domains; and fourth—revise and finalize the framework. Participants and procedures for each step are detailed below. Data collection began after the Institution Review Board determined the study was not human subjects’ research.

### Evaluate framework usability

#### Participants

The framework has been available on the healthcare organization’s website for the previous 7 years. The website provides a mechanism to submit an electronic form to request permission for use, which generates an automated email reply with the requested resources. We recruited individuals who had completed the online request for the original Iowa implementation framework. The list was cleaned of duplicates and inactive email addresses. An initial invitation to participate included an imbedded link to the survey. Respondents with unfinished surveys received automated reminder emails at 2 weeks and 1 month.

#### Procedures

We developed the survey to identify what aspects of the original implementation framework were useful. Participants were invited to complete an online survey using Qualtrics^XM^. The survey had sections exploring respondents’ use of and expertise with using the framework, evaluation of the framework (usability, generalizability, adaptability, accuracy, placement of strategies within the framework, and feedback on implementation phases and the target for each strategy), recommendations for updates and comments, and demographics. In the survey, we provided a list of the 81 names of the implementation strategies and asked respondents to select at least one of four phases in which each strategy should be used: (1) create awareness and interest, (2) build knowledge and commitment, (3) promote action and adoption, and (4) pursue integration and sustained use. The survey also queried respondents about seven potential new implementation strategies identified by the authors (i.e., advisory boards, cultural broker, disincentives, learning collaborative, revise professional roles, social media influencer, and visit other sites). These potential strategies were identified from reports about dissemination and implementation and experience leading EBP changes in the practice setting. Respondents were asked to recommend if any of the new implementation strategies should be added and, if so, the phase and target for each. They were also asked to suggest other new strategies. The survey took approximately 40 min to complete. See Supplemental Appendix A for the full survey.

We report frequencies and percentages to describe the demographics of the survey respondents and their beliefs about usability, likeliness to use in the future, and expertise with the framework. Cochran’s *Q* analyses were used to determine the difference in proportions for participants’ responses to the phases for each of 81 implementation strategies included in the survey. A *p*-value of 0.05 was used to determine if respondents were significantly more likely to place an implementation strategy in one of the four phases. Qualtrics^XM^ [48] was used for descriptive statistics and SAS [49] was used to compute Cochran’s *Q* analyses.

### Identify and specify strategies

#### Participants

An expert consensus panel was made up of three nurse researchers with expertise in the Iowa implementation framework, a nurse leader with clinical experience using and mentoring clinicians in the use of the implementation framework, and an implementation science expert with no prior experience with the Iowa implementation framework. The expert panel reviewed survey data about the strategies in the original framework, newly proposed strategies that survey participants considered and participants suggested additions, and determined 85 strategies to include in the pile sort method based on the strategies in the original framework, removing duplicates and separating discrete strategies and review of the survey results.

#### Procedures

To build consensus regarding revision, the expert panel reviewed survey results and discussed responses with focused attention on the phases and targets. This panel reviewed the innovation-decision process for individuals and organizations outlined in the Diffusion of Innovations [40] which provided the theoretical underpinnings for the consolidated four phases in the framework. Our process for operationalizing strategies included suggested elements created by Proctor et al. [31]. We reviewed a variety of additional theories, models, and frameworks (TMF). We then considered the description of elements for specifying individual implementation strategies [29–31, 33]. The panel created a crosswalk to outline previously developed strategy elements (i.e., title, phase, focus, definition, benefits, procedure, example, citations) [33, 50] and recommendations for specifying (i.e., name, temporality, action target, definition, outcome, action) [31] and missing specifications [31, 40, 51, 52]. We then created a template of constructs (i.e., name, phase, actor, target, definition, function, action procedure, considerations, clinician example, patient example, and citations) for specifying the implementation strategies included in the framework.

Our next step was to select the TMF best matching each specifying element to work synergistically with the Diffusion of Innovations theory [40]. From Proctor et al. [31], we specified elements including leadership, a key contextual factor for implementation [53–55], and the target or “who” or “where” the implementation strategy is directed. Constructs of the *Consolidated Framework for Implementation Research* [51] were used and provided a crosswalk to the Diffusion of Innovations theory [40, 51], while recognizing the systems perspective needed for implementation planning. We chose the Behavior Change Wheel [52] in order to include function as a specifying element to consider the link between each strategy and the related mechanism of action.

### Identify strategy domains

#### Participants

The expert panel recruited nurses in clinical nurse specialist and clinical nurse leader roles at our 800-bed quaternary academic medical center to inform the framework because of their vast clinical experience using it. A standing shared governance council meeting created opportunity to provide a description of the purpose and procedure, and a chance to volunteer. All 26 attendees at this meeting were recruited. Participants represented clinical areas and expertise across the health system and care of a wide variety of patient populations (e.g., ambulatory, emergency and trauma, intensive care, medical-surgical, and pediatrics).

#### Procedures

Cultural domain analysis [56, 57] was used as a similar methodology to stage 2 concept mapping described in the ERIC protocol [58] to capture additional user input. Participants were provided 85 cards, with one strategy name on each card, to sort into common categories. These cards did not include definitions of the strategies because we wanted the participants to rely on their own knowledge about each strategy. They were asked to put strategies into piles in whatever way made sense to them. The rules were to have more than one card in a pile and not put all cards in one pile. Each participant clipped each of their piles of cards together and placed all their piles in an envelope with an anonymous study identification number. A research assistant transcribed each participant’s pile sorting into a text document.

Data were entered into ANTHROPAC, a freely available domain analysis software program, and checks were run to ensure data entry accuracy. We also randomly selected three respondents’ pile sorts for a full review of data entry to check for accuracy. We then analyzed the data using multidimensional scaling to produce a domain map and Johnson’s hierarchical clustering matrix to visually display the clustering [59]. We cross referenced the two-dimensional domain map with Johnson’s hierarchical clustering matrix to define strategies clustering near each other on the map and having the closest association between each strategy in the matrix. For the implementation strategies that were not proximal on the map and did not cluster quantitatively with other strategies, we noted them as outliers for further discussion.

### Revise and finalize framework

#### Participants

The same expert panel re-convened to discuss study data and make final recommendations for the framework.

#### Procedures

First, we reviewed outliers from each of our methodologies. We discussed the three strategies survey respondents suggested adding to the framework (i.e., self-learning, gaming, and plan-do-study-act). We determined based on the literature these suggestions were not implementation strategies and so did not include them in the framework. For the outliers from the analysis of the two-dimensional domain map with Johnson’s hierarchical clustering matrix, we considered if they conceptually belonged in existing domains already identified (decisions described in more detail in the “Results” section). We also discussed implementation strategies that were identified after the survey and not included in the sorting exercise (patient input, patient decision aid, training, facilitator) and decided in which group they belonged. We reviewed the template of constructs for specifying implementation strategies. In preparation, we discussed the TMFs associated with each construct. We used these discussions to create a common understanding among the panel members, while remaining focused on keeping the implementation strategies discrete and actionable. One team member created a grid with each implementation strategy and associated constructs for the group to consider in specifying actor, function, target, and evaluation process measures for each discrete strategy. Group discussion centered on construct definitions, the form and function of implementation strategies, and resulted in a consensus for each strategy. The grid was also used to inform the review of the literature for each strategy and ongoing work to operationalize implementation strategies by updating or adding to a definition, procedure, considerations, and examples.

## Results

### Evaluate framework usability

We had 4059 requests for the original Iowa implementation framework which yielded 1578 active email addresses and 127 (8% response rate) completed the survey. Almost all (98%) were nurses, with 88% having at least a masters, and 84% from the USA (Table 1). The majority used the framework for an EBP change or student assignment (Table 1) and just 4% identified as a novice user (Table 1).

**Table 1:** Characteristics of survey respondents, *N* = 127

Characteristics	***N*** (%)
Discipline	
Nurse (includes nurse practitioner)	125 (98.4)
Physician	1 (0.8)
Others	1 (0.8)
Education	
Doctoral	70 (55.1)
Masters	50 (39.4)
Bachelors	6 (4.7)
Others	1 (0.8)
Current role	
Educator	41 (32.3)
Administrator	21 (16.5)
Clinician	16 (12.6)
Researcher	12 (9.5)
Student	3 (2.4)
Clinical nurse specialist/nurse leader	20 (15.8)
Others	14 (11.0)
Organization type	
Hospital	67 (52.3)
College or university	40 (31.3)
Ambulatory clinic	6 (4.7)
Community	3 (2.3)
Long-term skilled care	2 (1.6)
Others	10 (7.8)
Type of hospital	
Community	31 (44.3)
Academic medical center	19 (27.1)
Public (state or federal)	12 (17.1)
Critical access hospital	2 (2.9)
Others	6 (8.6)
Location	
USA	107 (84.3)
Asia/Pacific Islands	9 (7.1)
North America (non-US)	5 (3.9)
Middle East	4 (3.2)
Africa	1 (0.8)
Europe	1 (0.8)
Purpose for using the implementation model^a^	
Student paper or assignment	38 (18.2)
Organization EBP project	38 (18.2)
Unit/clinic EBP project	34 (16.3)
Classroom teaching	30 (14.4)
Research/grant	18 (8.6)
Publications/presentations	15 (7.2)
Magnet® submission	10 (4.8)
Others	6 (2.9)
Have not used	20 (9.6)
User rated experience with model	
Expert—extensive experience, highly skilled, confident, able to hone in on solutions	11(10.9)
Proficient—skilled, experienced, confident, able to troubleshoot problems	14 (13.9)
Competent—building skill and experience but confident in use	39 (38.6)
Advanced beginner—just beginning, developing experience and confidence	33 (32.7)
Novice—lacking experience and confidence	4 (4.0)

^a^Select all that apply—respondents could have selected more than one response to this item

**Fig. 3:**
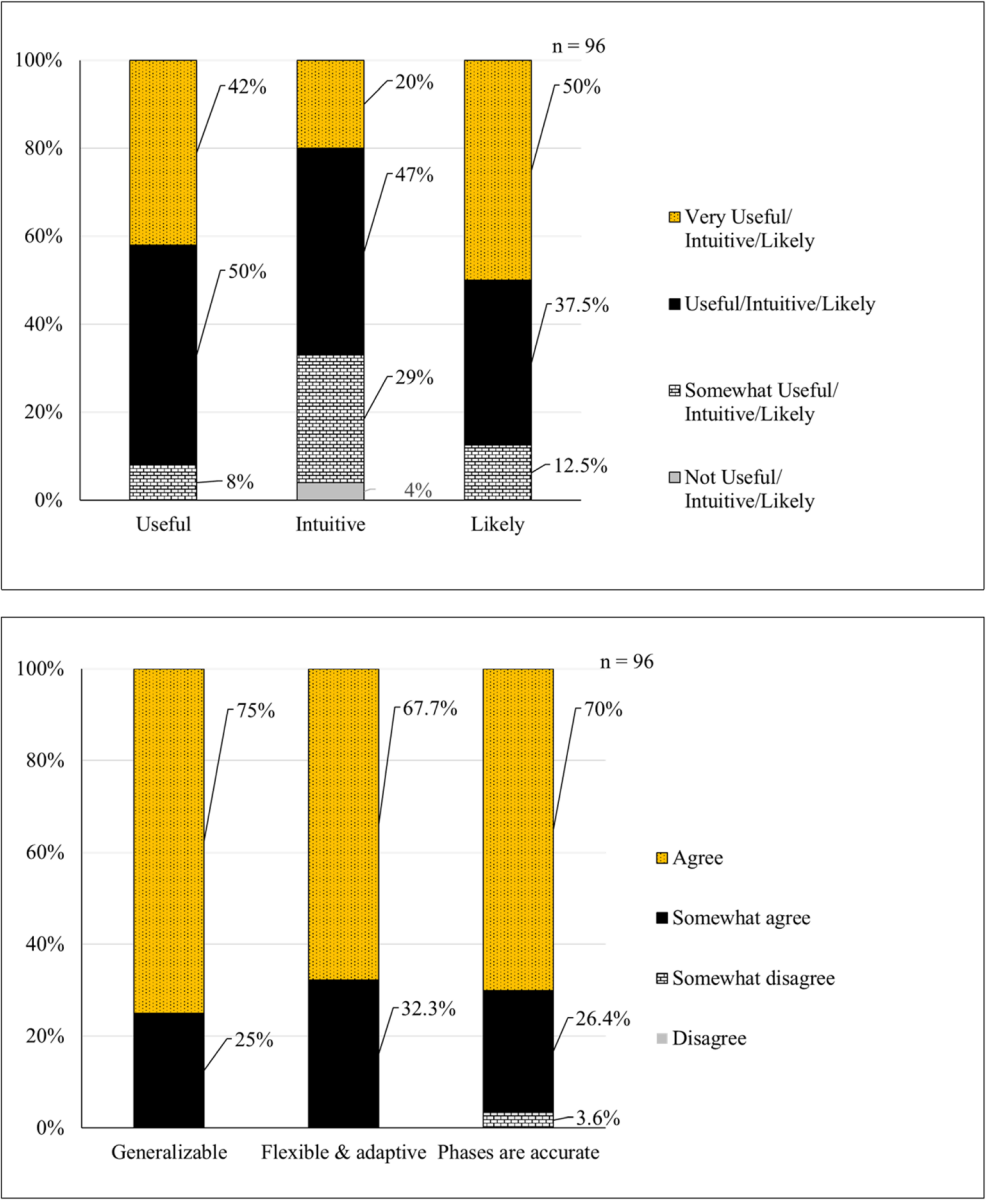
Usability evaluation

Most respondents found the original implementation framework as useful or very useful for EBP (92%), easy or very easy to use (71.9%), and intuitive or very intuitive for novice users (67%) (Fig. 3). All respondents (100%) agreed or somewhat agreed that the implementation framework is generalizable to different disciplines, settings, and populations and that the framework is flexible and adaptive to be used in conjunction with other EBP process models and frameworks. Most respondents (96.4%) agreed or somewhat agreed that the four phases accurately represented the stages of implementation. Nearly all agreed or somewhat agreed (98.2%) that the implementation framework contains a comprehensive selection of strategies. Lastly, 87.5% of respondents reported they were likely or very likely to use the implementation framework in the future.

Only 51 (40.2%) of survey respondents completed the section of the survey asking to select in which phase each implementation strategy belonged. Two-thirds of the 81 implementation strategies (*n* = 54, 66.7%) had a statistically significant agreement (*p* < 0.5) that the strategy has one primary phase (Table 2). Among those implementation strategies, half matched the phase on the original framework. Of the 54 strategies which respondents selected as having a statistically significant primary phase, 24 strategies (44.4%) did not have agreement between respondents and the original framework. Some of those strategies that were a mismatch between respondents and the original framework were local adaptation and simplify (*p* = 0.0006), make observable (0.044), and troubleshoot use (*p* <.0001). Respondents had no significant consensus identifying a primary phase for one-third of the strategies (*n* = 27, 33.3%). Some of these strategies were unit inservice (*p* = 0.174), unit newsletter (*p* = 0.932), posters or postings (*p* = 0.943), and unit orientation (*p* = 0.619) (Table 2). The expert panel reviewed the data, definition, and form and function for all strategies and identified phases to determine final placement.

**Table 2:** Respondents’ selections for which strategies belonged to a phase (*n* = 51), *n* (%)

Strategy number/name		Creating awareness and interest	Building knowledge and commitment	Promoting action and adoption	Pursuing integration and sustainability	*p*-value
01	Action plan	32 (62.8)	34 (66.7) §	**42 (82.4)** §	32 (62.8)	**0.034**
02	Actionable and timely data feedback	19 (37.3)	26 (51.0)	**34 (66.7)** §	32 (62.8)	**0.003**
03	*Advisory boards*					
04	Announcements and broadcasts	**42 (82.4)** §	25 (49.0)	25 (49.0)	22 (43.1)	**< 0.0001**
--	Annual report	23 (45.1)	16 (31.4)	16 (31.4)	**3 (64.7)** §	**0.0008**
05	Audit and feedback	21 (41.2)	21 (41.2)	31 (60.8)	**35 (68.6)** §	**0.003**
06	Audit key indicators	21 (41.2)	22 (43.1)	30 (58.8) §	**33 (64.7)**	**0.015**
07	Benchmark data	27 (52.9)	24 (47.1) §	30 (58.8)	**36 (70.6)**	**0.034**
08	Case studies	29 (56.9)	31(60.8) §	31 (60.8)	21 (41.2)	0.056
09	Celebrate local unit progress	23 (45.1)	25 (49.0)	30 (58.8)	**37 (72.6)** §	**0.004**
10	Change agents (e.g., change champion, core group, opinion leader, thought leader, etc.)	32 (62.8)	35 (68.6) §	34 (66.7) §	28 (54.9)	0.241
11	Checklist	19 (37.3)	22 (43.1)	**37 (72.6)** §	25 (49.0)	**0.0002**
12	Clinician input	27 (52.9)	35 (68.6) §	32 (62.8)	29 (56.9)	0.102
13	*Cultural broker*					
--	Competency metric for discontinuing training	16 (31.4)	20 (39.2)	18 (35.3)	24 (47.1) §	0.313
--	Continuing education programs	20 (39.2) §	**36 (70.6)**	26 (51.0)	28 (54.9)	**0.002**
14	Data collection by clinicians	19 (37.3)	24 (47.1)	**31 (60.8)** §	28 (54.9)	**0.032**
15	*Decision algorithm*					
16	Demonstrate workflow or decision algorithm	18 (35.3)	29 (56.9)	**34 (66.7)** §	30 (58.8)	**0.002**
17	*Disincentives*					
18	Disseminate credible evidence with clear implications for practice	25 (49.0)	22 (43.1) §	28 (54.9)	32 (62.8)	0.10
19	Distribute key evidence	22 (43.1) §	26 (51.0)	25 (49.0)	29 (56.9)	0.419
20	Documentation	15 (29.4)	17 (33.3)	25 (49.0) §	**26 (51.0)**	**0.014**
21	Education (e.g., live, virtual, or computer-based)	27 (52.9)	**35 (68.6)** §	28 (54.9)	22 (43.1)	**0.004**
22	Educational outreach or academic detailing	23 (45.1)	31 (60.8) §	25 (49.0) §	22 (43.1)	0.116
23	“Elevator speech”	**29 (56.9)**	26 (51.0)	20 (39.2) §	15 (29.4)	**0.001**
--	Financial incentives	16 (31.4)	13 (25.5)	22 (43.1)	**25 (49.0)** §	**0.017**
24	Focus groups for planning change	23 (45.1)	23 (45.1) §	28 (54.9)	22 (43.1)	0.477
25	*Gaming*					
26	Gap assessment/gap analysis	27 (52.9)	26 (51.0) §	25 (49.0)	18 (35.3)	0.135
27	Give evaluation results to colleagues	18 (35.3)	19 (37.3)	28 (54.9) §	**31 (60.8)**	**0.002**
28	Highlight advantages or anticipated impact	27 (52.9) §	27 (52.9)	26 (51.0)	25 (49.0)	0.939
29	Highlight compatibility	16 (31.4) §	21 (41.2)	23 (45.1)	18 (35.3)	0.332
30	Incentives	21 (41.2)	20 (39.2)	28 (54.9) §	25 (49.0)	0.215
31	Individual performance evaluation	15 (29.4)	17 (33.3)	**31 (60.8) **§	25 (49.0)	**0.0006**
32	Individualize data feedback	15 (29.4)	16 (31.4)	**29 (56.9)**	25 (49.0) §	**0.0016**
33	Inform organizational leaders	21 (41.2)	21 (41.2) §	25 (49.0)	24 (47.1)	0.692
34	Integrate practice change with other EBP protocols	13 (25.5)	20 (39.2) §	29 (56.9)	**30 (58.8)**	**0.0003**
35	*Interprofessional discussion and troubleshooting*					
36	Journal club	**27 (52.9)** §	32 (62.8)	21 (41.2)	12 (23.5)	**< .0001**
37	Knowledge broker(s)	26 (51.0) §	**30 (58.8)**	22 (43.1)	14 (27.5)	**0.001**
38	*Learning collaborative*					
39	Link practice change and power holder/stakeholder priorities	22 (43.1)	28 (54.9) §	28 (54.9)	25 (49.0)	0.373
40	Link to patient/family needs and organizational priorities	23 (45.1)	23 (45.1)	28 (54.9) §	25 (49.0)	0.521
41	Local adaptation and simplify	14 (27.5)	16 (31.4) §	**28 (54.9)**	25 (49.0)	**0.0006**
42	Make impact observable	19 (37.3)	21 (41.2) §	26 (51.0)	**29 (56.9)**	**0.044**
43	Match practice change with resources and equipment	12 (23.5)	16 (31.4) §	**27 (52.9)**	**27 (52.9)**	**0.0001**
44	Mobile “show on the road”	23 (45.1) §	20 (39.2)	21 (41.2)	22 (43.1)	0.861
--	Multidisciplinary discussion and troubleshooting	16 (31.4)	25 (49.0)	**30 (58.8)** §	27 (52.9)	**0.001**
45	Non-punitive discussion of results	13 (25.5)	15 (29.4)	25 (49.0) §	**26 (51.0)**	**0.001**
46	Patient decision aids	16 (31.4)	23 (45.1)	**28 (54.9)** §	22 (43.1)	**0.032**
47	Patient reminders	13 (25.5)	16 (31.4)	**26 (51.0)** §	21 (41.2)	**0.005**
--	Peer influence	24 (47.1)	24 (47.1)	26 (51.0)	22 (43.1) §	0.738
48	Personalize the messages to staff (e.g., reduces work, reduces infection exposure, etc.) based on actual improvement data	27 (52.9)	27 (52.9)	23 (45.1)	20 (39.2) §	0.322
49	Pocket guides	19 (37.3)	22 (43.1) §	**29 (56.9)**	24 (47.1)	**0.047**
50	*Positive deviance*					
51	Posters and postings/fliers	23 (45.1) §	25 (49.0)	24 (47.1)	23 (45.1)	0.943
--	Present in educational programs	25 (49.0)	**30 (58.8)**	28 (54.9)	18 (35.3) §	**0.008**
52	Project responsibility in unit or organizational committee	14 (27.5)	23 (45.1)	**27 (52.9)**	26 (51.0) §	**0.004**
53	Provide recognition at the point of care	15 (29.4)	20 (39.2)	25 (49.0) §	**26 (51.0)**	**0.016**
54	Public recognition	18 (35.3)	16 (31.4)	22 (43.1)	**27 (52.9)** §	**0.013**
55	Publicize new equipment	18 (35.3) §	16 (31.4)	**27 (52.9)**	24 (47.1)	**0.010**
56	Reminders or practice prompts	16 (31.4)	20 (39.2)	**29 (56.9)** §	26 (51.0)	**0.004**
57	Report into quality improvement program	11 (21.6)	15 (29.4)	**24 (47.1)** §	**25 (49.0)** §	**0.0007**
58	Report progress and updates	12 (23.5)	14 (27.5)	25 (49.0) §	**28 (54.9)**	**< .0001**
59	Report to senior leaders	11 (21.6)	12 (23.5) §	22 (43.1) §	**27 (52.9)** §	**0.0002**
60	Report within organizational infrastructure	14 (27.5)	18 (35.3) §	23 (45.1)	**28 (54.9)**	**0.005**
60	Resource manual or materials (i.e., electronic or hard copy)	15 (29.4)	24 (47.1) §	**28 (54.9)**	24 (47.1)	**0.011**
61	Resource materials and quick reference guides	17 (33.3)	25 (49.0)	**30 (58.8)** §	25 (49.0)	**0.010**
62	*Quick reference guide*					
63	Revise policy, procedure, or protocol	11 (21.6)	15 (29.4)	24 (47.1)	**28 (54.9)** §	**< .0001**
64	*Revise professional roles*					
65	Role model	18 (35.3)	23 (45.1)	**29 (56.9)** §	27 (52.9)	**0.008**
66	Rounding by unit and organizational leadership	23 (45.1)	21 (41.2)	29 (56.9) §	25 (49.0)	0.154
67	*Self-learning*					
68	Senior executives’ announcements	19 (37.3) §	14 (27.5)	21 (41.2)	22 (43.1)	0.207
69	Share protocol revisions with clinician that are based on feedback from clinicians, patient, or family	13 (25.5)	19 (37.3)	**27 (52.9)**	22 (43.1) §	**0.009**
70	*Simplify*					
71	Skill competence	11 (21.6)	23 (45.1)	**30 (58.8)** §	20 (39.2)	**< .0001**
72	Slogans and logos	25 (49.0) §	19 (37.3)	21 (41.2)	16 (31.4)	0.138
73	*Social media influencer*					
74	Sound bites	**26 (51.0)** §	16 (31.4)	20 (39.2)	16 (31.4)	**0.036**
75	Staff meetings	25 (49.0) §	24 (47.1)	26 (51.0)	23 (45.1)	0.842
76	Standing orders	9 (17.7)	13 (25.5)	**27 (52.9)** §	26 (51.0)	**< .0001**
--	Strategic plan	14 (27.5)	15 (29.4)	24 (47.1)	**26 (51.0)** §	**0.004**
77	Teamwork	26 (51.0)	29 (56.9) §	31 (60.8)	29 (56.9)	0.459
78	Trend results	16 (31.4)	18 (35.3)	**27 (52.9)**	24 (47.1) §	**0.025**
79	Troubleshoot use/application	9 (17.7)	21 (41.2) §	**28 (54.9)**	24 (47.1)	**< .0001**
80	Troubleshooting at the point of care/bedside	8 (15.7)	17 (33.3)	**27 (52.9)** §	26 (51.0)	**< .0001**
81	Try the practice change	11 (21.6)	17 (33.3)	**31 (60.8)** §	21 (41.2)	**< .0001**
82	Unit inservices	22 (43.1) §	27 (52.9)	29 (56.9)	23 (45.1)	0.174
83	Unit newsletter	25 (49.0) §	27 (52.9)	25 (49.0)	26 (51.0)	0.932
84	Unit orientation	22 (43.1)	24 (47.1)	24 (47.1) §	20 (39.2)	0.619
--	Update practice reminders	18 (35.3)	21 (41.2)	24 (47.1)	**28 (54.9)** §	**0.033**
85	*Visit other sites*					

Bold represents the most frequently selected phase and statistical significance at *p* < 0.05 using Cochran’s *Q* test

§Indicates which phase the strategy was listed in the original framework

The *p*-value examines whether there was a significant difference in responses between the phases. If the strategy does not have a strategy #, that strategy was not used for the card pile sort

Italicized strategies indicate the strategy was not used in the survey

### Identify and specify strategies

The template for this step in the study is shown in Table 3. In summary, the expert panel ended this step with 75 implementation strategies, thus reducing the total number of discrete strategies in the framework. Discussion led to strategies being unbundled (i.e., change agents became knowledge broker, opinion leader, change champion, etc.; posters and postings/flyers became two strategies—poster and flyer), simplified (incentive, financial incentive, and disincentive became incentive), and duplicates or redundancies being eliminated (i.e., audit, feedback, audit and feedback, and individual data feedback were revised to become audit indicators, data feedback to group, and data feedback to individual). Results of the specifying activity are available (Supplemental Table 4) and include phases into which the expert panel placed the strategies, as well as their domain, function, actor, and target.

**Table 3:** Implementation strategy specifications. This table provides descriptions for implementation strategy specifications or recommended specifying [31]

Implementation Strategy Specifications (Related Theory)		
**Name**		
Addresses ***what*** to call each implementation strategy to be able to identify and communicate those selected.		
**Phase(s)** [10, 33, 40]		
Addresses ***when*** to use each implementation strategy(ies).		
➭ Create Awareness & Interest	➭Promote Action & Adoption	
➭ Build Knowledge & Commitment	➭Pursue Integration & Sustained Use	
**Additional phases**		
Addresses additional options for ***when*** to use each implementation strategy(gies):		
**Domain(s)** [60, 61]		
Adds guidance for ***which*** implementation strategy(ies) to include.		
▪ Marketing	▪ Information	▪ Learning
▪ Commitment	▪ Change Agents	▪ Decision Support
▪ Adaptation	▪ Data	▪ Organizational Infrastructure
▪ Reinforcement		
**Definition**		
Provides detail for ***what*** each implementation strategy is addressing. It is not an operational definition (see action).		
**Function(s)** (43)		
Describes ***why*** the implementation strategy may work.		
▪ Education	▪ Modeling	▪ Coercion
▪ Enablement	▪ Incentivization	▪ Training
▪ Persuasion	▪ Restrictions	▪ Environmental restructuring
**Actor** (30)		
Identifies ***who*** could best provide this implementation strategy.		
▪ Payer	▪ Community stakeholders	▪ Outside consultants
▪ Clinicians	▪ Administrators	▪ Implementers (within organization)
▪ Intervention developers	▪ Patient	
**Target** (42)		
Describes ***where*** each implementation strategy will impact.		
▪ Intervention/localized protocol	▪ Characteristics of individuals	▪ Outer setting
▪ Process	▪ Inner setting	
**Action procedure**		
Provides directions for ***how to do*** each implementation strategy to improve their effectiveness.		
**Considerations**		
Addresses additional details for ***how to use*** each implementation strategy.		

### Identify strategy domains

**Fig. 4:**
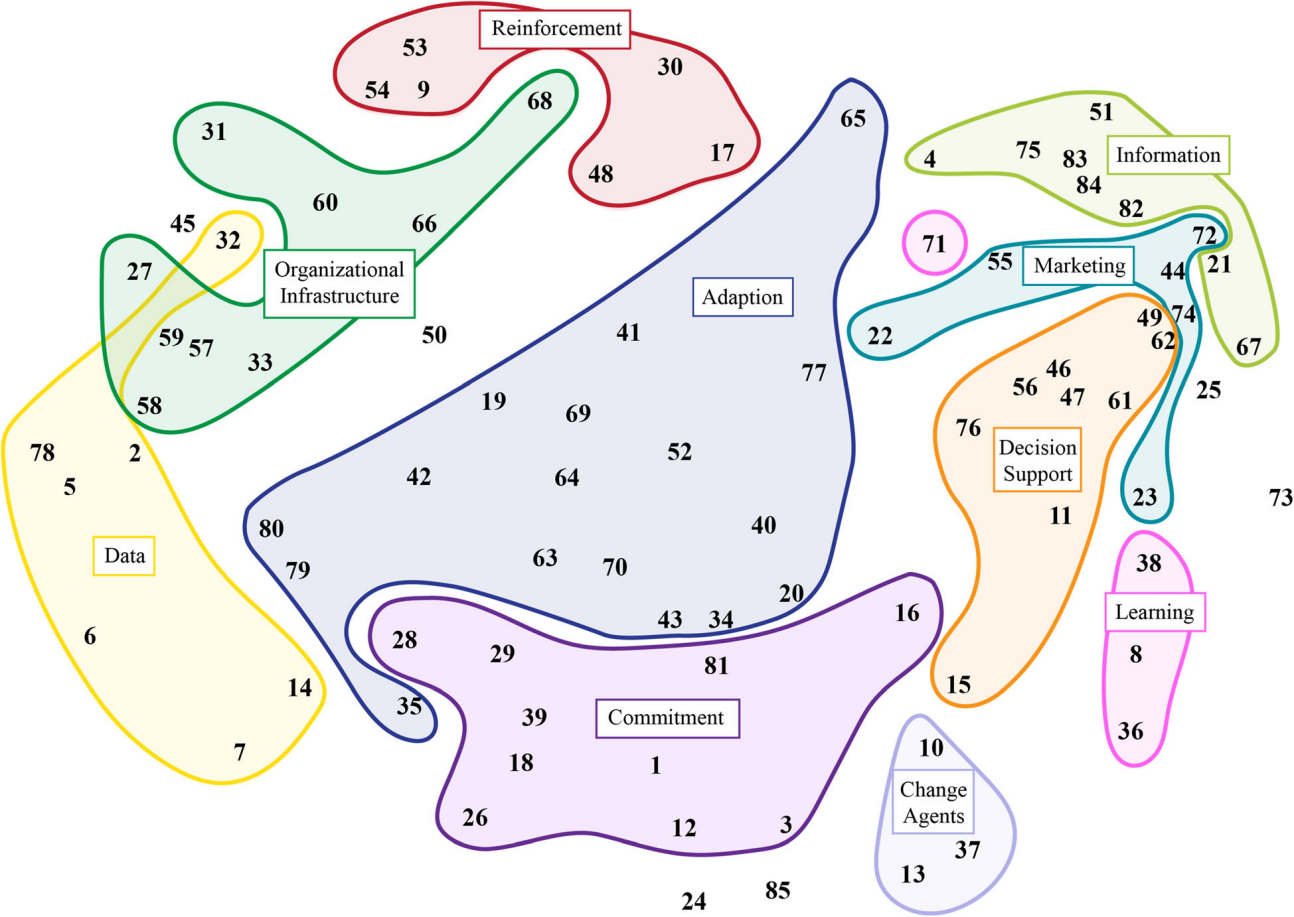
Domain mapping of implementation strategies identified by nurse leaders

**Fig. 5:**
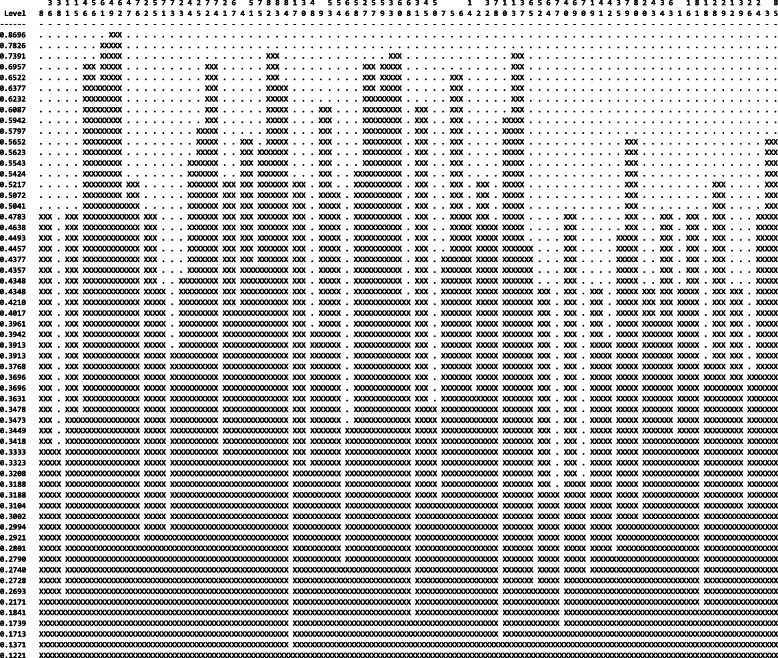
Domains of implementation strategies from Johnson’s hierarchical clustering

Among the attendees of the nurse specialists shared governance council, 26 participated and three were excluded for returning the cards as a single pile, resulting in 23 usable responses. Using the pile sorting methodology, our cultural domain analysis resulted in two visual displays of the nurse specialists group consensus regarding the categorization of implementation strategies (see Figs. 4 and 5). The expert panel used the domain map (Fig, 4) to identify domains of implementations strategies and referred to Johnson’s hierarchical clustering (Fig. 5) to help determine in which domain to include an implementation strategy, when strategies where on the border. This resulted in strategies being clustered into 10 domains. In addition, three of the strategies (i.e., skill competence, performance evaluation, and link to patient need) were outliers and did not fit meaningfully into the domains of either visual display. These were discussed individually.

### Revise and finalize framework

After the ten domains were determined, the expert panel independently reviewed and suggested labels for each domain. The expert panel considered the commonalities of the strategies and how the participants would think about each strategy and put them into action. Through the consensus process, we named the domains as follows: *Marketing*, *Information*, *Learning*, *Commitment*, *Change Agents*, *Decision Support*, *Adaptation*, *Data*, *Organizational Infrastructure*, and *Reinforcement*. Next, the 75 strategies were placed vertically in the primary phase of the implementation framework (identified in step 2), while keeping them horizontally within their domains. The result was a visual cascade of implementation strategies, within the four phases by domain.

For the three miscellaneous (outlier) and four added, unsorted implementation strategies, the expert panel individually reviewed the strategies and placed them in a related domain. They then met and formed a consensus regarding the domain for each strategy. Skill competence and training were placed into the *Learning* domain. Facilitator was placed in the *Change Agent* domain. Link to patient needs was placed in the *Commitment* domain. Patient decision aid was placed in the *Decision Support* domain. Patient input was placed in the *Adaptation* domain and performance evaluation was placed in the *Organizational Infrastructure* domain.

**Fig. 6:**
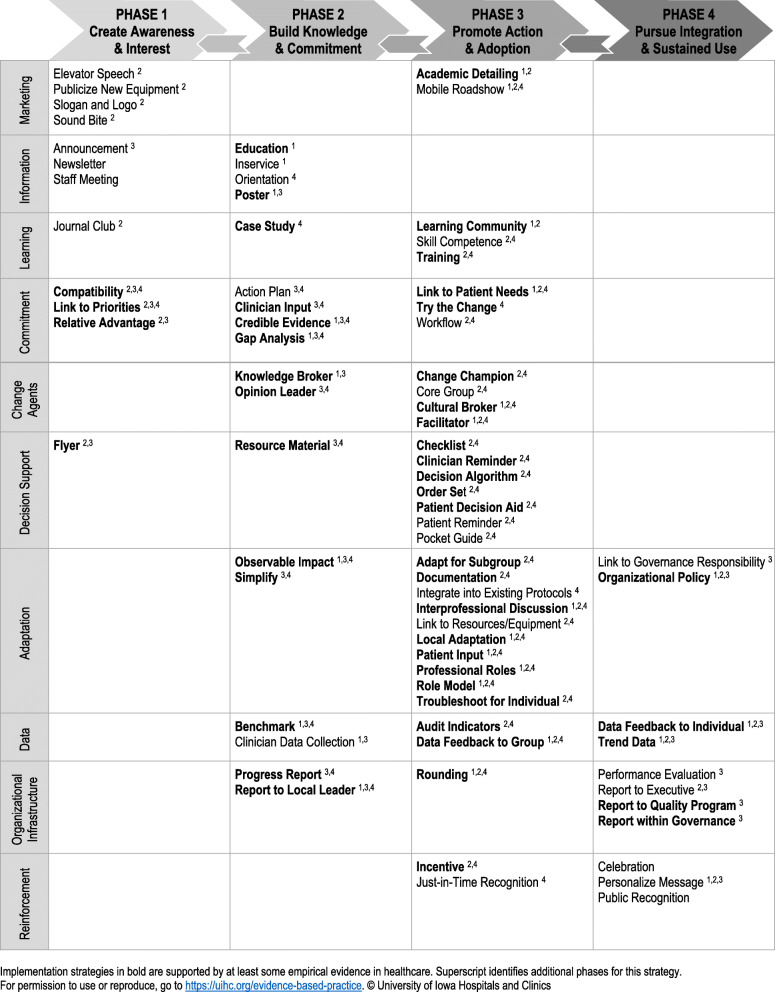
The Iowa Implementation for Sustainability Framework (IISF)

Finally, we discussed the design of the framework from a user perspective. To convey the iterative nature of the implementation step and the reality that team members are in different places relative to adoption (e.g., late adopter, new hires) while the team is making forward progress, we opted for arrows going forward through phases with an option to go back to other phases reflecting midstream corrections. We finalized the primary and other useful phases for each strategy. Each strategy was placed in the primary phase with superscripts for other phases in which a strategy could be useful. Strategies were clustered within their domain, as rows, when placed within their primary phase, as columns. To make strategies with at least some empirical evidence in healthcare stand out as potentially being more effective (as determined by literature reviews and content expertise by the first author), we used a bold type face. The expert panel then reviewed the two visualizations of the cultural domain analysis, finalized the implementation strategies in each domain, and labeled the domains. Lastly, we finalized the framework display and assigned a more descriptive name—Iowa Implementation for Sustainability Framework (Fig. 6).

## Discussion

The Iowa Implementation for Sustainability Frameworks (IISF) was developed based on user input and designed for clinicians to make implementation actionable, while promoting its use among researchers as a clinician-developed, clinician-facing framework. The IISF was originally designed based on the Diffusion of Innovations theory [40] and continues to reflect those theoretical underpinnings, while being adapted for application in practice. An important advantage is the IISF inclusion of the 75 discrete implementation strategies which offer a variety of options when planning implementation to avoid over-reliance on education and information sharing [13, 21, 62–64]. The framework phases and domains provide guidance on when to use strategies and suggest how to bundle them by crossing domains to address the cognitive, motivational, psychomotor, social, and organizational influences.

Implementation is a journey, not an event, and recognized to occur in phases over time [10, 40, 65–67]. The four phases originally adapted from the Diffusion of Innovations theory [40] include create awareness and interest, build knowledge and commitment, promote action and adoption, and finally pursue integration and sustained use. These four phases were confirmed by clinicians using the framework.

Phases for implementation differ from steps in the EBP process, though these have been confused [14, 15, 68]. Basic EBP process steps include identification of a need, determining fit of the identified need within the local context, creating a team, use of best evidence to determine practice recommendation, designing the EBP change, implementation, evaluation, sustain the change, and dissemination [8]. Phases are part of the implementation step, but often in reality of a non-linear practice change, both EBP process steps and implementation phases overlap (e.g., cycling between evaluation and implementation).

As implementation science develops, clarity is required in the language used to name implementation strategies and determinants used in TMF. Implementation strategies and frameworks should focus specifically on the implementation step within the EBP process and avoid mixing language relating implementation strategies (e.g., academic detailing, change agents, audit, and feedback), with project management (e.g., develop relationships, planning, organize team meetings, access funding, resource sharing agreement) and steps in the EBP process (e.g., create a clear purpose statement, create a team, identify practice recommendations, pilot/small test of change, implementation planning, evaluate). Our process for specifying also kept in mind the need to differentiate the use of effective implementation strategies from the evidence-based intervention, implementation process, and implementation outcomes [19, 27, 34, 35, 69–71]. This study and the 75 implementation strategies included in the IISF advance previous typologies by separating EBP process steps from implementation strategies.

Users were asked to identify the primary phase for each implementation strategy and were able to identify a primary phase for only two-thirds of the strategies. This may reflect the implementation strategy being useful and effective across phases (e.g., relative advantage). This may also reflect that users lack understanding of strategy names or inexperience with using the full range of implementation strategies available, despite these strategies having good evidence of effectiveness (e.g., academic detailing). Other strategies tend to be commonly used across the breadth of implementation phases and may represent a lack of guidance from and underdevelopment of the related mechanism of action. These strategies may be overused, and their effectiveness limited by relying too heavily on passive information sharing (e.g., distribute credible evidence, inservice, poster). These findings highlight the need for clear guidance and expertise to know how to bundle implementation strategies for a comprehensive, yet efficient implementation plan.

Grouping of strategies into domains offers an intermediary and explanatory step which may help to identify unique mechanisms of action [9, 72], and the associated implementation outcome [34, 37]. The domains identified in this framework may create a bridge to specifying implementation strategies and guiding use. These domains offer an added benefit when reporting results of systematic reviews of implementation strategies. Implementers and clinician leaders will benefit from the added explanatory details provided by the ten domains if research can evaluate and support the use of domains.

Understanding how implementation strategies work and the mechanism of action facilitates efficient and effective selection of implementation strategies, identifying strategy bundles, and importantly matching strategies to local setting’s contextual needs [19, 21, 73, 74]. Strategies must be selected to address local context needs [39] that cut across learning needs beyond cognitive and psychomotor skill development to build the EBP change into the team’s workflow [16, 75] and create new practice habits that can be sustained [76, 77].

In work based on the initial Iowa implementation framework, we identified the first known compilation of specified strategies for users [33, 50]. The current study and the IISF better align the names and specification of the strategies with other TMF. In addition to the elements specified by this study, we are currently reviewing literature and operationalizing each strategy by updating or adding to a definition, procedure, considerations, and examples for a full compilation of the IISF strategies in the next edition for the *Evidence-Based Practice in Action* monograph.

Still needed when specifying strategies is the development of methods to match local context with implementation strategy selection. Including the organizational perspective in adoption involves matching the local setting and the practice change [40]. Implementation science has focused on building organizational capacity [78–80]. To date, assessments of organizational needs and capacity building fall short of adding the final link required between the intervention developer and implementer with the clinician and patient as end users. The use of baseline evaluation of local data for implementation can provide guidance for matching implementation strategies with the local needs [8, 39]. Unlike implementation research frameworks for specifying outcome measures (e.g., RE-AIM) [31, 35, 81], the unique KABOB framework (i.e., Knowledge, Attitude, Behavior, Outcome, and Balancing Measures) for evaluation offers direct guidance from local assessment to select matching implementation strategies [39]. Research is needed to establish how best to create that critical match to local needs.

Actionable guidance and procedures to increase fidelity in use and reporting are needed. The expert panel took the first steps to develop this specification of implementation strategies for application in practice [29–31]. Discrete strategies require a name and conceptual definition and must be operationalized with further detail, so that each can be executed, measured, and compared in meaningful ways [31]. The strategy names were selected to be brief and, when possible, consistent with common use in practice and the implementation literature [31, 36, 38].

### Limitations

This study was needed to address updates in implementation science. The IISF is intended to be useful for clinicians across a range of expertise. Yet, survey respondents were highly educated and may not reflect novice users’ perspectives. While feedback from more experienced users may have been well informed, additional insights from novice users would strengthen the usefulness of the IISF as a resource for EBP. Another limitation is nurse leaders who completed the pile sorts and the expert consensus panel were all from the same institution; however, the strength of this approach was the depth of knowledge of the framework and it was representative of the culture of the institution. In addition, the domains created from the cultural domain analysis were not returned to the committee members for review. The COVID pandemic and surge needs for patient care took precedent. Thus, their confirmation of those findings is missing. Participants were almost exclusively nurses. While additional evaluation by other interprofessional team members is warranted, we believe a study on the perspectives of nurses working at the point of care is a strength. Nurses who provide direct care to patients are the ones who need implementation strategies in their work and thus implementation science should be informed by them. Additionally, our response rate (8%) was low; thus, those who responded may have more favorable perceptions than those who did not respond. Those who requested the original implementation guide may also not have used the guide so they may not have had an opinion.

Also missing from the framework is the patient perspective [38]. The next step is co-design with engagement and empowering patient’s role in implementation [82]. To that end, additional work is needed to operationalize implementation strategies for patients to be drivers of EBP improvements and implementation strategy use. Currently, the IISF has some strategies focused on patients (i.e., patient decision aid) and the team is identifying patient-driven examples (e.g., asthma action plan); this continues to be a gap for implementation science to fill.

Preliminary anecdotal feedback has been positive when the IISF has been reported in research presentation and workshops using the IISF. Early input indicates the framework is more visually appealing, less dense, and easy to interpret after minimal orientation to phases, domains, and implementation strategies [60, 61]. Further evaluation and validation are warranted. Use will be tracked through the same online automated permission request portal [83], creating an opportunity for future research.

## Conclusion

This study reduces gaps between conduct of research and application in EBP critical to quality healthcare. Through a multi-step iterative process, this study evaluates and begins to validate and strengthen the previous Iowa implementation framework to become the Iowa Implementation for Sustainability Framework (IISF). The four implementation phases and 75 distinctive implementation strategies in the IISF were identified. The targets in the original Iowa implementation framework were focused on people and systems; the structure of the IISF shifted to include 10 newly identified domains that are indicative of the mechanism of action. The external validity of these domains has yet to be established.

Standard use of strategy names is foundational to compare and understand what implementation strategies are being used and when they are effective, in what dose, for which topics, by whom, and in what context. Implementation and the Iowa implementation framework have evolved over three decades as a step in the Iowa Model of EBP which has primarily been used by nurses but has broad applications for any interprofessional team [8, 10, 84–86]. The IISF is now more effective as a framework because it (1) offers implementation strategy names that are discrete and actionable, while remaining distinct to the implementation step within the EBP process; and (2) provides a structure that is usable by a novice or expert and offers a typology to guide nurses, interprofessional teams, and researchers as they strive to efficiently implement and sustain evidence-based improvements in healthcare.

This study builds upon an implementation framework widely used in nursing. There is a need to bridge the silos currently reflected in implementation research. Nursing has been on the forefront and that early research is largely missing from current developments, resulting in reinvention and rediscovery when the needs in healthcare have become even more pressing. We challenge public health and medicine to look at nursing research and TMF, and we challenge nursing to look at the implementation science literature when trying to select strategies for change. It is time for nursing to model this interprofessional and international work and bring the nursing perspective into presentations at international implementation and dissemination conferences and publish in interprofessional and international journals.

The IISF is designed to be application oriented and lead to effective implementation planning using actionable implementation strategies. Establishing standard and descriptive names for discrete strategies is warranted to promote comparison and determine core elements of associated action procedures. Additional work is needed to determine if these domains guide bundling of implementation strategies to improve implementation outcomes—adoption, sustained use, and cost.

## Supplementary Information

**Additional file 1:** Survey: Evaluation of the Evidence-Based Implementation Model.

**Additional file 2:** Table 4. Implementation strategies selected by expert panel on second step of study.

## Data Availability

The datasets used and/or analyzed during the current study are available from the corresponding author on reasonable request.

## References

[1] Ament SM, de Groot JJ, Maessen JM, Dirksen CD, van der Weijden T, Kleijnen J (2015). Sustainability of professionals’ adherence to clinical practice guidelines in medical care: a systematic review. BMJ Open..

[2] Birken SA, Haines ER, Hwang S, Chambers DA, Bunger AC, Nilsen P (2020). Advancing understanding and identifying strategies for sustaining evidence-based practices: a review of reviews. Implement Sci..

[3] Hailemariam M, Bustos T, Montgomery B, Barajas R, Evans LB, Drahota A (2019). Evidence-based intervention sustainability strategies: a systematic review. Implement Sci..

[4] Lennox L, Linwood-Amor A, Maher L, Reed J (2020). Making change last? Exploring the value of sustainability approaches in healthcare: a scoping review. Health Res Policy Syst..

[5] Nadalin Penno L, Davies B, Graham ID, Backman C, MacDonald I, Bain J, Johnson AM, Moore J, Squires J (2019). Identifying relevant concepts and factors for the sustainability of evidence-based practices within acute care contexts: a systematic review and theory analysis of selected sustainability frameworks. Implement Sci..

[6] Wiltsey Stirman S, Kimberly J, Cook N, Calloway A, Castro F, Charns M (2012). The sustainability of new programs and innovations: a review of the empirical literature and recommendations for future research. Implement Sci..

[7] Fernandez ME, Ten Hoor GA, van Lieshout S, Rodriguez SA, Beidas RS, Parcel G (2019). Implementation mapping: using intervention mapping to develop implementation strategies. Front Public Health..

[8] Iowa Model Collaborative (2017). Iowa model of evidence-based practice: revisions and validation. Worldviews Evid Based Nurs..

[9] Cane J, Richardson M, Johnston M, Ladha R, Michie S (2015). From lists of behaviour change techniques (BCTs) to structured hierarchies: comparison of two methods of developing a hierarchy of BCTs. Br J Health Psychol..

[10] Cullen L, Adams SL (2012). Planning for implementation of evidence-based practice. J Nurs Adm..

[11] Lyon AR, Cook CR, Locke J, Davis C, Powell BJ, Waltz TJ (2019). Importance and feasibility of an adapted set of implementation strategies in schools. J Sch Psychol..

[12] Michie S, Richardson M, Johnston M, Abraham C, Francis J, Hardeman W, Eccles MP, Cane J, Wood CE (2013). The behavior change technique taxonomy (v1) of 93 hierarchically clustered techniques: building an international consensus for the reporting of behavior change interventions. Ann Behav Med..

[13] Moreno EM, Moriana JA (2016). User involvement in the implementation of clinical guidelines for common mental health disorders: a review and compilation of strategies and resources. Health Res Policy Syst..

[14] Powell BJ, McMillen JC, Proctor EK, Carpenter CR, Griffey RT, Bunger AC (2012). A compilation of strategies for implementing clinical innovations in health and mental health. Med Care Res Rev..

[15] Powell BJ, Waltz TJ, Chinman MJ, Damschroder LJ, Smith JL, Matthieu MM, Proctor EK, Kirchner JAE (2015). A refined compilation of implementation strategies: results from the Expert Recommendations for Implementing Change (ERIC) project. Implement Sci..

[16] Scholl I, LaRussa A, Hahlweg P, Kobrin S, Elwyn G (2018). Organizational- and system-level characteristics that influence implementation of shared decision-making and strategies to address them - a scoping review. Implement Sci..

[17] Titler MG, Conlon P, Reynolds MA, Ripley R, Tsodikov A, Wilson DS (2016). The effect of a translating research into practice intervention to promote use of evidence-based fall prevention interventions in hospitalized adults: a prospective pre-post implementation study in the U.S.. Appl Nurs Res.

[18] Titler MG, Everett LQ (2001). Translating research into practice. Considerations for critical care investigators. Crit Care Nurs Clin North Am..

[19] Waltz TJ, Powell BJ, Fernandez ME, Abadie B, Damschroder LJ (2019). Choosing implementation strategies to address contextual barriers: diversity in recommendations and future directions. Implement Sci..

[20] Tomasone JR, Kauffeldt KD, Chaudhary R, Brouwers MC (2020). Effectiveness of guideline dissemination and implementation strategies on health care professionals’ behaviour and patient outcomes in the cancer care context: a systematic review. Implement Sci..

[21] Lewis CC, Klasnja P, Powell BJ, Lyon AR, Tuzzio L, Jones S, Walsh-Bailey C, Weiner B (2018). From classification to causality: advancing understanding of mechanisms of change in implementation science. Front Public Health..

[22] Colquhoun H, Leeman J, Michie S, Lokker C, Bragge P, Hempel S (2014). Towards a common terminology: a simplified framework of interventions to promote and integrate evidence into health practices, systems, and policies. Implement Sci..

[23] Curran GM, Bauer M, Mittman B, Pyne JM, Stetler C (2012). Effectiveness-implementation hybrid designs: combining elements of clinical effectiveness and implementation research to enhance public health impact. Med Care..

[24] Larsen KR, Michie S, Hekler EB, Gibson B, Spruijt-Metz D, Ahern D, Cole-Lewis H, Ellis RJB, Hesse B, Moser RP, Yi J (2017). Behavior change interventions: the potential of ontologies for advancing science and practice. J Behav Med..

[25] Powell BJ, Fernandez ME, Williams NJ, Aarons GA, Beidas RS, Lewis CC, McHugh SM, Weiner BJ (2019). Enhancing the impact of implementation strategies in healthcare: a research agenda. Front Public Health..

[26] Strifler L, Barnsley JM, Hillmer M, Straus SE (2020). Identifying and selecting implementation theories, models and frameworks: a qualitative study to inform the development of a decision support tool. BMC Med Inform Decis Mak..

[27] Thompson GN, Estabrooks CA, Degner LF (2006). Clarifying the concepts in knowledge transfer: a literature review. J Adv Nurs..

[28] Kirchner J, Waltz T, Powell BJ, Smith J, Proctor E, Brownson RC, Colditz GA, Proctor EK (2018). Implementation strategies. Dissemination and implementation research in health: translating science to practice.

[29] Leeman J, Birken SA, Powell BJ, Rohweder C, Shea CM (2017). Beyond “implementation strategies”: classifying the full range of strategies used in implementation science and practice. Implement Sci..

[30] Perry CK, Damschroder LJ, Hemler JR, Woodson TT, Ono SS, Cohen DJ (2019). Specifying and comparing implementation strategies across seven large implementation interventions: a practical application of theory. Implement Sci..

[31] Proctor EK, Powell BJ, McMillen JC (2013). Implementation strategies: recommendations for specifying and reporting. Implement Sci..

[32] Michie S, Carey RN, Johnston M, Rothman AJ, de Bruin M, Kelly MP, Connell LE (2018). From theory-inspired to theory-based interventions: a protocol for developing and testing a methodology for linking behaviour change techniques to theoretical mechanisms of action. Ann Behav Med..

[33] Cullen L, Hanrahan K, Farrington M, DeBerg J, Tucker S, Kleiber C (2018). Evidence-based practice in action: comprehensive strategies, tools and tips from the University of Iowa Hospitals and Clinics.

[34] Donaldson NE, Rutledge DN, Ashley J (2004). Outcomes of adoption: measuring evidence uptake by individuals and organizations. Worldviews Evid Based Nurs..

[35] Proctor E, Silmere H, Raghavan R, Hovmand P, Aarons G, Bunger A, Griffey R, Hensley M (2011). Outcomes for implementation research: conceptual distinctions, measurement challenges, and research agenda. Adm Policy Ment Health..

[36] Rudd BN, Davis M, Beidas RS (2020). Integrating implementation science in clinical research to maximize public health impact: a call for the reporting and alignment of implementation strategy use with implementation outcomes in clinical research. Implement Sci..

[37] Sumner JA, Carey RN, Michie S, Johnston M, Edmondson D, Davidson KW (2018). Using rigorous methods to advance behaviour change science. Nat Hum Behav..

[38] Cotterill S, Knowles S, Martindale AM, Elvey R, Howard S, Coupe N, Wilson P, Spence M (2018). Getting messier with TIDieR: embracing context and complexity in intervention reporting. BMC Med Res Methodol..

[39] Cullen L, Hanrahan K, Tucker SJ, Gallagher-Ford L (2019). Data-driven precision implementation approach. Am J Nurs..

[40] Rogers EM (2003). Diffusion of innovations.

[41] Beeler C, Kerley D, Davis C, Hazen D, Snyderman W, Lyons K, Sadowski J, Sweeney J, Dbeibo L, Kelley K, Webb DH (2019). Strategies for the successful implementation of disinfecting port protectors to reduce CLABSI in a large tertiary care teaching hospital. Am J Infect Control..

[42] Cerderbom S, Bjerk M, Bergland A (2020). The tensions between micro-, meso- and macro-levels: physiotherapists’ views of their role towards fall prevention in the community - a qualitative study. BMC Health Serv Res..

[43] Chiwaula CH, Kanjakaya P, Chipeta D, Chikatipwa A, Kalimbuka T, Zyambo L, Nkata S, Jere DL (2021). Introducing evidence based practice in nursing care delivery, utilizing the Iowa model in intensive care unit at Kamuzu Central Hospital. Malawi. Int J Africa Nurs Sci..

[44] Downey J, Kruse D, Plonczynski DJ (2019). Nurses reduce epidural-related urinary retention and postpartum hemorrhages. J Perianesth Nurs..

[45] Pramita Sari RD, Rokhanawati D (2020). How far is the implementation of evidence-based practice in midwifery care?. Int J Adv Sci Technol..

[46] Salcido ME, Monsivais DB (2016). Screening and management of overweight and obesity at a university student health center. Nurse Pract..

[47] Speroni KG, McLaughlin MK, Freisen MA (2020). Use of evidence-based practice models and research findings in Magnet-designated hospitals across the United States: national survey results. Worldviews Evid Based Nurs..

[48] Qualtrics^XM^. Qualtrics^XM^ software Provo, UT: Qualtrics; n.d. Available from: https://www.qualtrics.com. Accessed 4 Sept 2019.

[49] SAS Institute Inc. SAS software Cary, NC: SAS Institute, inc. Available from: https://www.sas.com/en_us/company-information.html. Accessed 3 Mar 2020.

[50] Cullen L, Hanrahan K, Tucker S, Rempel G, Jordan K (2012). Evidence-based practice building blocks: comprehensive strategies, tools and tips.

[51] Damschroder LJ, Hagedorn HJ (2011). A guiding framework and approach for implementation research in substance use disorders treatment. Psychol Addict Behav..

[52] Michie S, van Stralen MM, West R (2011). The behaviour change wheel: a new method for characterising and designing behaviour change interventions. Implement Sci..

[53] Allen JD, Towne SD, Maxwell AE, DiMartino L, Leyva B, Bowen DJ, Linnan L, Weiner BJ (2017). Measures of organizational characteristics associated with adoption and/or implementation of innovations: a systematic review. BMC Health Serv Res..

[54] Gifford WA, Squires JE, Angus DE, Ashley LA, Brosseau L, Craik JM, Domecq MC, Egan M, Holyoke P, Juergensen L, Wallin L, Wazni L, Graham ID (2018). Managerial leadership for research use in nursing and allied health care professions: s systematic review. Implement Sci..

[55] Li SA, Jeffs L, Barwick M, Stevens B (2018). Organizational contextual features that influence the implementation of evidence-based practices across healthcare settings: a systematic integrative review. Syst Rev..

[56] Borgatti S (1994). Cultural domain analysis. J Quant Anthrop..

[57] Weller SC, Romney AK (1988). Systematic data collection.

[58] Waltz TJ, Powell BJ, Chinman MJ, Smith JL, Matthieu MM, Proctor EK, Damschroder LJ, Kirchner JAE (2014). Expert recommendations for implementing change (ERIC): protocol for a mixed methods study. Implement Sci..

[59] Kruskal JB, Wish M (1978). Multidimensional scaling.

[60] Cullen L, Edmonds S, Hanrahan K, Wagner M. Precision implementation approach workshop. Iowa: 2021 National Evidence-Based Practice Conference; 2021.

[61] Cullen C, Hanrahan K, Edmonds S, Reisinger H, Wagner, M. A study to determine external validity of the Iowa implementation for sustainability framework. [Paper presentation]. 13^th^ Annual Conference of the Science of Dissemination and Implementation in Health, Academy Health, Washington, D.C. (virtual). 2020.

[62] Beard E, West R, Lorencatto F, Gardner B, Michie S, Owens L, Shahab L (2019). What do cost-effective health behaviour-change interventions contain? A comparison of six domains. PLoS One..

[63] Häggman-Laitila A, Mattila LR, Melender HL (2017). A systematic review of the outcomes of educational interventions relevant to nurses with simultaneous strategies for guideline implementation. J Clin Nurs..

[64] Wu Y, Brettle A, Zhou C, Ou J, Wang Y, Wang S (2018). Do educational interventions aimed at nurses to support the implementation of evidence-based practice improve patient outcomes? A systematic review. Nurse Educ Today..

[65] Aarons GA, Hurlburt M, Horwitz SM (2011). Advancing a conceptual model of evidence-based practice implementation in public service sectors. Adm Policy Ment Health..

[66] Grol R, Wensing M, Eccles M, Davis D (2013). Improving patient care: the implementation of change in health care.

[67] Prochaska JO, DiClemente CC, Norcross JC (1992). In search of how people change. Applications to addictive behaviors. Am Psychol..

[68] Bunger AC, Powell BJ, Robertson HA, MacDowell H, Birken SA, Shea C (2017). Tracking implementation strategies: a description of a practical approach and early findings. Health Res Policy Syst.

[69] Eldh AC, Almost J, DeCorby-Watson K, Gifford W, Harvey G, Hasson H, Kenny D, Moodie S, Wallin L, Yost J (2017). Clinical interventions, implementation interventions, and the potential greyness in between - a discussion paper. BMC Health Serv Res..

[70] Powell BJ, Stanick CF, Halko HM, Dorsey CN, Weiner BJ, Barwick MA, Damschroder LJ, Wensing M, Wolfenden L, Lewis CC (2017). Toward criteria for pragmatic measurement in implementation research and practice: a stakeholder-driven approach using concept mapping. Implement Sci..

[71] Rapport F, Clay-Williams R, Churruca K, Shih P, Hogden A, Braithwaite J (2018). The struggle of translating science into action: foundational concepts of implementation science. J Eval Clin Pract..

[72] Cane J, O'Connor D, Michie S (2012). Validation of the theoretical domains framework for use in behaviour change and implementation research. Implement Sci..

[73] Bohlen LC, Michie S, de Bruin M, Rothman AJ, Kelly MP, Groarke HNK, Carey RN, Hale J, Johnston M (2020). Do combinations of behavior change techniques that occur frequently in interventions reflect underlying theory?. Ann Behav Med..

[74] Powell BJ, Beidas RS, Lewis CC, Aarons GA, McMillen JC, Proctor EK (2017). Methods to improve the selection and tailoring of implementation strategies. J Behav Health Serv Res..

[75] Jiang V, Brooks EM, Tong ST, Heintzman J, Krist AH (2020). Factors influencing uptake of changes to clinical preventive guidelines. J Am Board Fam Med..

[76] Arsenault Knudsen EN, King BJ, Steege LM (2021). The realities of practice change: nurses’ perceptions. J Clin Nurs..

[77] Dawson A, Henriksen B, Cortvriend P (2019). Guideline implementation in standardized office workflows and exam types. J Prim Care Community Health..

[78] Brownson RC, Fielding JE, Green LW (2018). Building capacity for evidence-based public health: reconciling the pulls of practice and the push of research. Annu Rev Public Health..

[79] Cullen L, Hanrahan K, Farrington M, Anderson R, Dimmer E, Miner R, Suchan T, Rod E (2020). Evidence-based practice change champion program improves quality care. J Nurs Adm..

[80] Ehrhart MG, Aarons GA, Farahnak LR (2014). Assessing the organizational context for EBP implementation: the development and validity testing of the Implementation Climate Scale (ICS). Implement Sci..

[81] Glasgow RE, Vogt TM, Boles SM (1999). Evaluating the public health impact of health promotion interventions: the RE-AIM framework. Am J Public Health..

[82] Boaz A, Robert G, Locock L, Sturmey G, Gager M, Vougioukalou S, Ziebland S, Fielden J (2016). What patients do and their impact on implementation. J Health Organ Manag..

[83] University of Iowa Hospitals & Clinics. Complimentary resources Iowa City, IA: Office of Nursing Research and Evidence-Based Practice, Department of Nursing Services and Patient Care, University of Iowa Hospitals & Clinics;. n.d. Available from: https://uihc.org/evidence-based-practice.

[84] Titler MG, Kleiber C, Steelman V, Goode C, Rakel B, Barry-Walker J, Small S, Buckwalter K (1994). Infusing research into practice to promote quality care. Nurs Res..

[85] Titler MG, Kleiber C, Steelman VJ, Rakel BA, Budreau G, Everett LQ, Buckwalter KC, Tripp-Reimer T, Goode CJ (2001). The Iowa Model of evidence-based practice to promote quality care. Crit Care Nurs Clin North Am..

[86] Watson CA, Bulechek GM, McCloskey JC (1987). QAMUR: a quality assurance model using research. J Nurs Qual Assur..

