# Supplementary material for: Iowa Implementation for Sustainability Framework
Source: Implement Sci. 2022 Jan 4;17:1. doi: 10.1186/s13012-021-01157-5 (PMC8725573; doi:10.1186/s13012-021-01157-5)
Supplement: Supplementary file 1 — Additional file 1: Survey: Evaluation of the Evidence-Based Implementation Model. [file 13012_2021_1157_MOESM1_ESM.pdf]

UNIVERSITY OF IOWA HEALTH CARE

Default Question Block

Evaluation of the Evidence-Based Practice Implementation Model

Background

Thank you for agreeing to participate in this survey. Your expert opinion is highly valued. The questions should take approximately 30 minutes to complete. If you partially complete the survey you may recommence it at any time from the same computer.

There are numerous process models that describe the necessary steps for evidence-based practice in nursing. 'Implementation' is a critical step in each of these models, yet there has traditionally been limited guidance on how best this is achieved. The Evidence-Based Practice Implementation Model (implementation model) was first published in 2012 as an application-oriented resource for nurses to plan evidence-based practice implementation in complex healthcare systems. The implementation model aides in the selection of implementation strategies that are suitable to the phase of implementation and specific target groups.

The implementation phases are displayed as columns progressing from awareness and interest to integration and sustained use. Each column includes strategies based on the goal for that implementation phase. Implementation strategies also target two distinct groups and are arranged in rows accordingly. The first row specifically targets the clinicians and organizational leaders, including key stakeholders. The second row builds support for the practice change at the organization or systems level. Project leaders select implementation strategies for their EBP initiative that are appropriate for their particular unit and organization.

Click below to open Implementation Strategies in a new window
Implementation Strategies for EBP

Figure 8.2 Implementation Strategies for Evidence-Based Practice

	Create Awareness & Interest	Build Knowledge & Commitment	Promote Action & Adoption	Pursue Integration & Sustained Use
Connecting With Clinicians, Organizational Leaders, and Key Stakeholders	■ Highlight advantages* or anticipated impact*<br>■ Highlight compatibility*<br>■ Continuing education programs*<br>■ Sound bites*<br>■ Journal club*<br>■ Slogans & logos<br>■ Staff meetings<br>■ Unit newsletter<br>■ Unit inservices<br>■ Distribute key evidence<br>■ Posters and postings/fliers<br>■ Mobile 'show on the road'<br>■ Announcements & broadcasts	■ Education (e.g., live, virtual, or computer-based)*<br>■ Pocket guides<br>■ Link practice change & power holder/stakeholder priorities*<br>■ Change agents (e.g., change champion*, core group*, opinion leader*, thought leader, etc.)<br>■ Educational outreach or academic detailing*<br>■ Integrate practice change with other EBP protocols*<br>■ Disseminate credible evidence with clear implications for practice*<br>■ Make impact observable*<br>■ Gap assessment/gap analysis*<br>■ Clinician input*<br>■ Local adaptation* & simplify*<br>■ Focus groups for planning change*<br>■ Match practice change with resources & equipment<br>■ Resource manual or materials (i.e., electronic or hard copy)<br>■ Case studies	■ Educational outreach/academic detailing*<br>■ Reminders or practice prompts*<br>■ Demonstrate workflow or decision algorithm<br>■ Resource materials and quick reference guides<br>■ Skill competence*<br>■ Give evaluation results to colleagues*<br>■ Incentives*<br>■ Try the practice change*<br>■ Multidisciplinary discussion & troubleshooting<br>■ "Elevator speech"<br>■ Data collection by clinicians<br>■ Report progress & updates<br>■ Change agents (e.g., change champion*, core group*, opinion leader*, thought leader, etc.)<br>■ Role model*<br>■ Troubleshooting at the point of care/bedside<br>■ Provide recognition at the point of care*	■ Celebrate local unit progress*<br>■ Individualize data feedback*<br>■ Public recognition*<br>■ Personalize the messages to staff (e.g., reduces work, reduces infection exposure, etc.) based on actual improvement data<br>■ Share protocol revisions with clinician that are based on feedback from clinicians, patient, or family<br>■ Peer influence<br>■ Update practice reminders
Building Organizational System Support	■ Knowledge broker(s)<br>■ Senior executives announcements<br>■ Publicize new equipment	■ Teamwork*<br>■ Troubleshoot use/application*<br>■ Benchmark data*<br>■ Inform organizational leaders*<br>■ Report within organizational infrastructure*<br>■ Action plan*<br>■ Report to senior leaders	■ Audit key indicators*<br>■ Actionable and timely data feedback*<br>■ Non-punitive discussion of results*<br>■ Checklist*<br>■ Documentation*<br>■ Standing orders*<br>■ Patient reminders*<br>■ Patient decision aids*<br>■ Rounding by unit & organizational leadership*<br>■ Report into quality improvement program*<br>■ Report to senior leaders<br>■ Action plan*<br>■ Link to patient/family needs & organizational priorities<br>■ Unit orientation<br>■ Individual performance evaluation	■ Audit and feedback*<br>■ Report to senior leaders*<br>■ Report into quality improvement program*<br>■ Revise policy, procedure, or protocol*<br>■ Competency metric for discontinuing training<br>■ Project responsibility in unit or organizational committee<br>■ Strategic plan*<br>■ Trend results*<br>■ Present in educational programs<br>■ Annual report<br>■ Financial incentives*<br>■ Individual performance evaluation

After seven years of use it is time to evaluate the Evidence-Based Practice Implementation Models usefulness, validity and impact on adoption of EBP. This survey seeks feedback on the implementation model from users to ascertain its strengths, limitations and content validity.

The following questions relate to you and your experience using the Evidence-Based Practice Implementation Model (implementation model).

What is your professional discipline?

- Dietitian
- Dentist
- Nurse
- Occupational Therapist
- Pharmacist
- Physical Therapist
- Physician
- Psychologist
- Social Worker
- Speech-Language Pathologist

- ○ [ ] Other:

What is your highest level of education?

- ○ Associate Degree
- ○ Bachelor Degree
- ○ Master Degree
- ○ Doctoral Degree
- ○ [ ] Other:

What is your primary role in your current organization?

- ○ Clinician
- ○ Administrator
- ○ Educator
- ○ Researcher
- ○ Clinical Nurse Specialist/Practice Leader
- ○ Student
- ○ Not currently employed
- ○ [ ] Other:

What type of organization do you currently work for?

- ○ College or University
- ○ Hospital
- ○ Long term skilled care
- ○ Community
- ○ Ambulatory clinic
- ○ [ ] Other:

What type of hospital do you work for? [select all that apply]:

- ☐ Public (state or federal) facility
- ☐ Academic medical center
- ☐ Community hospital
- ☐ Critical access hospital
- ☐ [ ] Other:

What type of community organization do you work for? [select all that apply]:

- ☐ Home care
- ☐ Public health
- ☐ School
- ☐ Hospice
- ☐ [ ] Other:

What is the location of your organization?

- ○ Africa
- ○ Asia/ Pacific
- ○ Europe
- ○ Latin America
- ○ Middle East
- ○ United States
- ○ North America (non-US)

In which state do you currently reside?

[▾]

What was your purpose for using the implementation model? (select all that apply)

- [ ] Classroom teaching
- [ ] Student paper or assignment
- [ ] Magnet application/reapplication
- [ ] Organization wide EBP project
- [ ] Unit/clinic based EBP project
- [ ] Research/ research grant
- [ ] Publication/presentation
- [ ] I have not used it
- [ ] ____________ Other:

How did you use the implementation model? (select all that apply)

- [ ] As a standalone implementation model
- [ ] In conjunction with the Iowa Model of Evidence-Based Practice
- [ ] In conjunction with another model for EBP
- [ ] ____________ Other

Which model did you use in conjunction with another model for EBP?

How would you rate your experience with the implementation model?

- ○ Novice- lacking experience and confidence
- ○ Advanced Beginner- just beginning, developing experience and confidence
- ○ Competent- building skill and experience but confident in use
- ○ Proficient- skilled, experienced, confident, able to troubleshoot problems
- ○ Expert- extensive experience, highly skilled, confident, able to hone in on solutions

The following questions seek your opinion on the usefulness, usability, generalizability, and adaptability of the implementation model.

How useful do you find the implementation model as a resource for EBP?

- ○ Not useful
- ○ Somewhat useful
- ○ Useful
- ○ Very useful

What changes would you recommend to increase the implementation models usefulness?

How easy do you find the implementation model to use?

- ○ Not easy
- ○ Somewhat easy
- ○ Easy
- ○ Very easy

What changes would you recommend to increase the implementation models ease of use?

How intuitive is the implementation model to novice users?

- Not intuitive
- Somewhat intuitive
- Intuitive
- Very intuitive

What changes would you recommend to increase ease of use for novice users?

Do you believe the model is generalizable to different disciplines, settings, and populations?

- Disagree
- Somewhat disagree
- Somewhat agree
- Agree

What changes would you recommend to make the model more generalizable?

Do you believe the model is flexible and adaptive enough to be used in conjunction with other EBP models and frameworks?

- Disagree
- Somewhat disagree
- Somewhat agree
- Agree

What changes would you recommend to make the model more flexible and adaptive?

How likely are you to use the implementation model in the future?

- Not likely
- Somewhat likely
- Likely
- Very likely

What changes would you recommend that would increase the likelihood you would use it again?

The following questions seek your opinion on the components of the implementation model (the implementation strategies, implementation phases, and socio-ecological levels).

Do you believe the implementation model phases (creating awareness and interest, building knowledge and commitment, promoting action and adoption, and pursuing integration and sustainability) accurately represent the stages of implementation?

- Disagree
- Somewhat disagree
- Somewhat agree
- Agree

What changes would you recommend to the phases of the implementation model?

Do you believe that the target groups identified in the implementation model (clinicians and organizational leaders, including key stakeholders; and organizational and systems support) accurately represent the socio-ecological levels associated with implementation?

- Disagree
- Somewhat disagree
- Somewhat agree
- Agree

What changes would you recommend to the target groups?

Would adding a target group for patients/care givers be an important socio-ecological level to add?

- Yes
- No

Do you believe the implementation model contains a comprehensive selection of implementation strategies?

- Disagree
- Somewhat disagree
- Somewhat agree
- Agree

What changes would you recommend to the implementation strategies?

Block 1

For each implementation strategy please select all of the phase(s) and target group(s) that you believe the strategy can be applied to.

	Phase (select all that apply)					Target Group [select all that apply]			Comment
	Creating awareness and interest	Building knowledge and commitment	Promoting action and adoption	Pursuing integration and sustainability	Other	Practitioners, organizational leaders, key stakeholders	Organizational and systems support	Other	Enter comments below
Action plan	☐	☐	☐	☐	☐	☐	☐	☐	

	Phase (select all that apply)					Target Group [select all that apply]			Comment
	Creating awareness and interest	Building knowledge and commitment	Promoting action and adoption	Pursuing integration and sustainability	Other	Practitioners, organizational leaders, key stakeholders	Organizational and systems support	Other	Enter comments below
Actionable and timely data feedback	☐	☐	☐	☐	☐	☐	☐	☐	
Announcements and broadcasts	☐	☐	☐	☐	☐	☐	☐	☐	
Annual report	☐	☐	☐	☐	☐	☐	☐	☐	
Audit and feedback	☐	☐	☐	☐	☐	☐	☐	☐	
Audit key indicators	☐	☐	☐	☐	☐	☐	☐	☐	
Benchmark data	☐	☐	☐	☐	☐	☐	☐	☐	
Case studies	☐	☐	☐	☐	☐	☐	☐	☐	
Celebrate local unit progress	☐	☐	☐	☐	☐	☐	☐	☐	
	Creating awareness and interest	Building knowledge and commitment	Promoting action and adoption	Pursuing integration and sustainability	Other	Practitioners, organizational leaders, key stakeholders	Organizational and systems support	Other	Enter comments below
Change agents (e.g., change champion, core group, opinion leader, thought leader)	☐	☐	☐	☐	☐	☐	☐	☐	
Checklist	☐	☐	☐	☐	☐	☐	☐	☐	
Clinician input	☐	☐	☐	☐	☐	☐	☐	☐	
Competency metric for discontinuing training	☐	☐	☐	☐	☐	☐	☐	☐	
Continuing education programs	☐	☐	☐	☐	☐	☐	☐	☐	
Data collection by clinicians	☐	☐	☐	☐	☐	☐	☐	☐	
Demonstrate workflow or decision algorithm	☐	☐	☐	☐	☐	☐	☐	☐	
Disseminate credible evidence with clear implications for practice	☐	☐	☐	☐	☐	☐	☐	☐	
Distribute key evidence	☐	☐	☐	☐	☐	☐	☐	☐	
	Creating awareness and interest	Building knowledge and commitment	Promoting action and adoption	Pursuing integration and sustainability	Other	Practitioners, organizational leaders, key stakeholders	Organizational and systems support	Other	Enter comments below
Documentation	☐	☐	☐	☐	☐	☐	☐	☐	
Education (e.g., live, virtual, or computer-based)	☐	☐	☐	☐	☐	☐	☐	☐	
Educational outreach/academic detailing	☐	☐	☐	☐	☐	☐	☐	☐	
Elevator speech	☐	☐	☐	☐	☐	☐	☐	☐	
Financial incentives	☐	☐	☐	☐	☐	☐	☐	☐	
Focus groups for planning change	☐	☐	☐	☐	☐	☐	☐	☐	
Gap assessment/gap analysis	☐	☐	☐	☐	☐	☐	☐	☐	
Give evaluation results to colleagues	☐	☐	☐	☐	☐	☐	☐	☐	
Highlight advantages or anticipated impact	☐	☐	☐	☐	☐	☐	☐	☐	
	Creating awareness and interest	Building knowledge and commitment	Promoting action and adoption	Pursuing integration and sustainability	Other	Practitioners, organizational leaders, key stakeholders	Organizational and systems support	Other	Enter comments below
Highlight compatibility	☐	☐	☐	☐	☐	☐	☐	☐	
Incentives	☐	☐	☐	☐	☐	☐	☐	☐	
Individual performance evaluation	☐	☐	☐	☐	☐	☐	☐	☐	
Individualize data feedback	☐	☐	☐	☐	☐	☐	☐	☐	

	Phase (select all that apply)					Target Group [select all that apply]			Comment
	Creating awareness and interest	Building knowledge and commitment	Promoting action and adoption	Pursuing integration and sustainability	Other	Practitioners, organizational leaders, key stakeholders	Organizational and systems support	Other	Enter comments below
Inform organizational leaders	☐	☐	☐	☐	☐	☐	☐	☐	
Integrate practice change with other EBP protocols	☐	☐	☐	☐	☐	☐	☐	☐	
Journal club	☐	☐	☐	☐	☐	☐	☐	☐	
Knowledge broker	☐	☐	☐	☐	☐	☐	☐	☐	
Link practice change and power holder/stakeholder priorities	☐	☐	☐	☐	☐	☐	☐	☐	
	Creating awareness and interest	Building knowledge and commitment	Promoting action and adoption	Pursuing integration and sustainability	Other	Practitioners, organizational leaders, key stakeholders	Organizational and systems support	Other	Enter comments below
Link to patient/family needs and organizational priorities	☐	☐	☐	☐	☐	☐	☐	☐	
Local adaptation and simplify	☐	☐	☐	☐	☐	☐	☐	☐	
Make impact observable	☐	☐	☐	☐	☐	☐	☐	☐	
Match practice change with resources and equipment	☐	☐	☐	☐	☐	☐	☐	☐	
Mobile “show on the road”	☐	☐	☐	☐	☐	☐	☐	☐	
Multidisciplinary discussion and troubleshooting	☐	☐	☐	☐	☐	☐	☐	☐	
Non-punitive discussion of results	☐	☐	☐	☐	☐	☐	☐	☐	
Patient decision aids	☐	☐	☐	☐	☐	☐	☐	☐	
Patient reminders	☐	☐	☐	☐	☐	☐	☐	☐	
	Creating awareness and interest	Building knowledge and commitment	Promoting action and adoption	Pursuing integration and sustainability	Other	Practitioners, organizational leaders, key stakeholders	Organizational and systems support	Other	Enter comments below
Peer influence	☐	☐	☐	☐	☐	☐	☐	☐	
Personalize the messages to staff (e.g., reduces work, reduces infection exposure) based on actual improvement data	☐	☐	☐	☐	☐	☐	☐	☐	
Pocket guides	☐	☐	☐	☐	☐	☐	☐	☐	
Posters and postings/fliers	☐	☐	☐	☐	☐	☐	☐	☐	
Present in educational programs	☐	☐	☐	☐	☐	☐	☐	☐	
Project responsibility in unit or organizational committee	☐	☐	☐	☐	☐	☐	☐	☐	
Provide recognition at the point of care	☐	☐	☐	☐	☐	☐	☐	☐	
Public recognition	☐	☐	☐	☐	☐	☐	☐	☐	
Publicize new equipment	☐	☐	☐	☐	☐	☐	☐	☐	
	Creating awareness and interest	Building knowledge and commitment	Promoting action and adoption	Pursuing integration and sustainability	Other	Practitioners, organizational leaders, key stakeholders	Organizational and systems support	Other	Enter comments below
Reminders or practice prompts	☐	☐	☐	☐	☐	☐	☐	☐	
Report into quality improvement program	☐	☐	☐	☐	☐	☐	☐	☐	
Report progress and updates	☐	☐	☐	☐	☐	☐	☐	☐	
Report to senior leaders	☐	☐	☐	☐	☐	☐	☐	☐	

	Phase (select all that apply)					Target Group [select all that apply]			Comment
	Creating awareness and interest	Building knowledge and commitment	Promoting action and adoption	Pursuing integration and sustainability	Other	Practitioners, organizational leaders, key stakeholders	Organizational and systems support	Other	Enter comments below
Report within organizational infrastructure	☐	☐	☐	☐	☐	☐	☐	☐	
Resource manual or materials	☐	☐	☐	☐	☐	☐	☐	☐	
Resource materials and quick reference guides	☐	☐	☐	☐	☐	☐	☐	☐	
Revise policy, procedure, or protocol	☐	☐	☐	☐	☐	☐	☐	☐	
Role model	☐	☐	☐	☐	☐	☐	☐	☐	
	Creating awareness and interest	Building knowledge and commitment	Promoting action and adoption	Pursuing integration and sustainability	Other	Practitioners, organizational leaders, key stakeholders	Organizational and systems support	Other	Enter comments below
Rounding by unit and organizational leadership	☐	☐	☐	☐	☐	☐	☐	☐	
Senior executive announcements	☐	☐	☐	☐	☐	☐	☐	☐	
Share protocol revisions with clinicians that are based on feedback from clinicians, patients, or family	☐	☐	☐	☐	☐	☐	☐	☐	
Skill competence	☐	☐	☐	☐	☐	☐	☐	☐	
Slogans and logos	☐	☐	☐	☐	☐	☐	☐	☐	
Sound bites	☐	☐	☐	☐	☐	☐	☐	☐	
Staff meetings	☐	☐	☐	☐	☐	☐	☐	☐	
Standing orders	☐	☐	☐	☐	☐	☐	☐	☐	
Strategic plan	☐	☐	☐	☐	☐	☐	☐	☐	
	Creating awareness and interest	Building knowledge and commitment	Promoting action and adoption	Pursuing integration and sustainability	Other	Practitioners, organizational leaders, key stakeholders	Organizational and systems support	Other	Enter comments below
Teamwork	☐	☐	☐	☐	☐	☐	☐	☐	
Trend results	☐	☐	☐	☐	☐	☐	☐	☐	
Troubleshoot use/application	☐	☐	☐	☐	☐	☐	☐	☐	
Troubleshooting at the point of care/bedside	☐	☐	☐	☐	☐	☐	☐	☐	
Trying the practice change	☐	☐	☐	☐	☐	☐	☐	☐	
Unit in-services	☐	☐	☐	☐	☐	☐	☐	☐	
Unit newsletter	☐	☐	☐	☐	☐	☐	☐	☐	
Unit orientation	☐	☐	☐	☐	☐	☐	☐	☐	
Update practice reminders	☐	☐	☐	☐	☐	☐	☐	☐	

Should the strategies listed below be added to the model?

	Yes	No
Advisory boards	☐	☐
Cultural broker	☐	☐
Disincentives	☐	☐
Learning collaborative	☐	☐
Revise professional roles	☐	☐
Social media influencer	☐	☐
Visit other sites	☐	☐

For each new implementation strategy you chose, please select all of the phase(s) and target group(s) that you believe the strategy can be applied to.

	Phase [select all that apply]					Target Group [select all that apply]			Click to write Column 3
	Creating awareness and interest	Building knowledge and commitment	Promoting action and adoption	Pursuing integration and sustainability	Other	Practitioners, organizational leaders, key stakeholders	Organizational and systems support	Other	Enter comments below
» Advisory boards	☐	☐	☐	☐	☐	☐	☐	☐	
» Cultural broker	☐	☐	☐	☐	☐	☐	☐	☐	
» Disincentives	☐	☐	☐	☐	☐	☐	☐	☐	
» Learning collaborative	☐	☐	☐	☐	☐	☐	☐	☐	
» Revise professional roles	☐	☐	☐	☐	☐	☐	☐	☐	
» Social media influencer	☐	☐	☐	☐	☐	☐	☐	☐	
» Visit other sites	☐	☐	☐	☐	☐	☐	☐	☐	

Please list any additional implementation strategies you recommend and select all of the phase(s) and target group(s) that you believe the strategy can be applied to.

	Phase [select all that apply]					Target Group [select all that apply]			Comment
	Creating awareness and interest	Building knowledge and commitment	Promoting action and adoption	Pursuing integration and sustainability	Other	Practitioners, organizational leaders, key stakeholders	Organizational and systems support	Other	Enter comments below
Additional implementation strategies:	☐	☐	☐	☐	☐	☐	☐	☐	
Click to write Statement 2	☐	☐	☐	☐	☐	☐	☐	☐	
Click to write Statement 3	☐	☐	☐	☐	☐	☐	☐	☐	
Click to write Statement 4	☐	☐	☐	☐	☐	☐	☐	☐	
Click to write Statement 5	☐	☐	☐	☐	☐	☐	☐	☐	
Click to write Statement 6	☐	☐	☐	☐	☐	☐	☐	☐	

Block 2

Thank you for participating in this survey. If you would like to leave any additional comments, please do so below.

Powered by Qualtrics
