# Supplementary material for: Iowa Implementation for Sustainability Framework
Source: Implement Sci. 2022 Jan 4;17:1. doi: 10.1186/s13012-021-01157-5 (PMC8725573; doi:10.1186/s13012-021-01157-5)
Supplement: Supplementary file 2 — Additional file 2: Table 4. Implementation strategies selected by expert panel on second step of study. [file 13012_2021_1157_MOESM2_ESM.docx]

**Table 4 (Supplement).** Implementation strategies selected by expert panel on second step of study

| **Strategy Number/Name**  Cullen et al., 2020 | | **Best Match to Intended Phase Purpose** | | | | **Phases where strategy may be useful** | | | | **Domain**  Cullen et al., 2020 | **Function**  Michie et al., 2011 | **Actor**  Proctor et al., 2013 | **Target**  Damschroder & Hagedorn, 2011 |
| --- | --- | --- | --- | --- | --- | --- | --- | --- | --- | --- | --- | --- | --- |
|  |  | **Phases**  Rogers, 2003 | | | | **Phases**  Rogers, 2003 | | | |  |  |  |  |
|  |  | **1** | **2** | **3** | **4** | **1** | **2** | **3** | **4** |  |  |  |  |
| 1 | Action Plan |  | X |  |  |  | X | X | X | Commitment | Enablement | Implementers, outside consultant | Inner setting |
| 2 | Announcement | X |  |  |  | X |  | X |  | Information | Persuasion | Administrators, community stakeholders, intervention developers | Inner setting |
| 3 | Case studies |  | X |  |  |  | X |  | X | Learning | Education | Implementers, clinicians, patients | Individual |
| 4 | Celebration |  |  |  | X |  |  |  | X | Reinforcement | Incentivisation | Administrators, clinicians, patients, community stakeholders, implementers | Inner setting |
| 5 | Just-in-time Recognition |  |  | X |  |  |  | X | X | Reinforcement | Persuasion | Clinicians | Individual, Inner setting |
| 6 | Public recognition |  |  |  | X |  |  |  | X | Reinforcement | Persuasion | Administrators, clinicians, patients, community stakeholders, implementers, intervention developers | Inner setting, Outer setting |
| 7 | Opinion Leader |  | X |  |  |  | X | X | X | Change agent | Persuasion | Clinicians, community stakeholder, intervention developers | Inner setting |
| 8 | Change Champion |  |  | X |  |  | X | X | X | Change agent | Enablement | Clinicians, patient, community stakeholder | Inner setting |
| 9 | Knowledge Broker |  | X |  |  | X | X | X |  | Change agent | Enablement | Outside consultants, intervention developers | Inner setting, Outer setting |
| 10 | Facilitator |  |  | X |  | X | X | X | X | Change agent | Enablement | Implementer, outside consultant | Inner setting, Outer setting |
| 11 | Thought leader | X |  |  |  | X |  |  |  | Change agent | Persuasion | Intervention developer, clinician, community stakeholders | Individual |
| 12 | Core Group |  |  | X |  |  | X | X | X | Change agent | Persuasion | Implementers, clinicians, patients, community stakeholders | Inner setting |
| 13 | Cultural Broker |  |  | X |  | X | X | X | X | Change agent | Environmental restructuring | Clinicians, community stakeholders | Inner setting |
| 14 | Patient input |  |  | X |  | X | X | X | X | Patient input | Environmental restructuring | Patient, community stakeholders | Outer setting |
| 15 | Checklist |  |  | X |  |  | X | X | X | Decision support | Environmental restructuring | Implementers, clinicians | Inner setting |
| 16 | Clinician Input |  | X |  |  |  | X | X | X | Commitment | Environmental restructuring | Clinicians | Inner setting |
| 17 | Audit indicators |  |  | X |  |  | X | X | X | Data | Education | Payer, administrator, outside consultant, implementer | Inner setting |
| 18 | Data feedback to group |  |  | X |  | X | X | X | X | Data | Persuasion | Implementers, clinicians, patients, outside consultants, community stakeholders | Inner setting |
| 19 | Data feedback to individualize |  |  |  | X | X | X | X | X | Data | Restriction | Administrators, clinician | Individual |
| 20 | Benchmark |  | X |  |  | X | X | X | X | Data | Coercion | Payers, administrators, implementers, clinicians, outside consultants | Outer setting |
| 21 | Clinician data collection |  | X |  |  | X | X | X |  | Data | Education | Clinicians | Individual |
| 22 | Trend data |  |  |  | X | X | X | X | X | Data | Education | Payers, administrators, implementers, clinicians, outside consultants | Inner setting, Outer setting |
| 23 | Gap analysis |  | X |  |  | X | X | X | X | Commitment | Persuasion | Payers, administrators, implementers, clinicians, outside consultants | Individual |
| 24 | Workflow |  |  | X |  |  | X | X | X | Commitment | Environmental restructuring | Implementers, clinicians | Inner setting |
| 25 | Decision Algorithm |  |  | X |  |  | X | X | X | Decision Support | Environmental restructuring | Implementers, clinicians, intervention developers | Inner setting |
| 26 | Credible Evidence |  | X |  |  | X | X | X | X | Commitment | Education | Intervention developers, implementers, outside consultants, clinicians, patients, payers | Individual |
| 27 | Documentation |  |  | X |  |  | X | X | X | Adaptation | Environmental restructuring | Implementers, clinicians, administrators, patients, intervention developers | Inner setting |
| 28 | Order set |  |  | X |  |  | X | X | X | Decision support | Environmental restructuring | Clinicians, implementers, intervention developers | Inner setting |
| 29 | Education |  | X |  |  | X | X |  |  | Information | Education | Clinicians, patients, implementers, intervention developers | Individual |
| 30 | Training |  |  | X |  |  | X | X | X | Learning | Training | Implementers, clinicians, patients | Individual |
| 31 | Academic Detailing |  |  | X |  | X | X | X |  | Marketing | Persuasion | Implementers, clinicians, outside consultant, administrators, intervention developers | Individual |
| 32 | Relative advantage | X |  |  |  | X | X | X |  | Commitment | Persuasion | Implementers, clinicians, patients, payers, administrators, outside consultants, intervention developers, community stakeholder | Intervention |
|  |  |  |  |  |  |  |  |  |  |  |  |  |  |
|  |  |  |  |  |  |  |  |  |  |  |  |  |  |
|  |  |  |  |  |  |  |  |  |  |  |  |  |  |
| 33 | Compatibility | X |  |  |  | X | X | X | X | Commitment | Persuasion | Implementers, clinicians, patients, administrators, payers, intervention developers, community stakeholder | Inner setting |
| 34 | Observable impact |  | X |  |  | X | X | X | X | Adaptation | Incentivisation | Patients, clinicians, implementers, outside consultants, intervention developers | Intervention |
| 35 | Local Adaptation |  |  | X |  | X | X | X | X | Adaptation | Enablement | Clinicians, patients, implementers, outside consultants, community stakeholder | Intervention |
| 36 | Try the change |  |  | X |  |  |  | X | X | Commitment | Enablement | Clinicians, patients, implementers, outside consultants, community stakeholder | Inner setting |
| 37 | Simplify |  | X |  |  |  | X | X | X | Adaptation | Enablement | Implementers, clinicians, patients, intervention developers, outside consultants, administrator | Intervention |
| 38 | Elevator Speech | X |  |  |  | X | X |  |  | Marketing | Persuasion | Implementer, clinician, patient, community stakeholder, outside consultant, administrator | Outer setting, inner setting |
| 39 | Incentives (include disincentive) |  |  | X |  |  | X | X | X | Reinforcement | Incentivisation | Payer, administrator, implementers, community stakeholders, outside consultant | Inner setting |
| 40 | Performance evaluation |  |  |  | X |  |  | X | X | Organizational infrastructure | Restriction | Administrator, clinicians | Individual, inner setting |
| 41 | Report to executive |  |  |  | X |  | X | X | X | Organizational infrastructure | Environment restructuring | Implementers, clinicians, patients, community stakeholders, outside consultants | Inner setting, Outer setting |
| 42 | Report – within governance |  |  |  | X |  |  | X | X | Organizational infrastructure | Restriction | Clinicians, implementers, patients, community stakeholders | Inner setting |
| 43 | Report to quality program |  |  |  | X |  |  | X | X | Organizational Infrastructure | Enablement | Clinicians, implementers, patients, community stakeholders | Inner setting |
| 44 | Progress report |  | X |  |  |  | X | X | X | Organizational infrastructure | Persuasion | Implementers, clinicians, patients | Inner setting, individuals |
| 45 | Report to local leaders |  | X |  |  | X | X | X | X | Organizational infrastructure | Incentivisation, environmental restructuring, persuasion | Implementers, clinicians, patients | Inner setting |
| 46 | Integrate into existing protocols |  |  | X |  |  |  | X | X | Adaptation | Restriction | Implementers, clinicians, patients | Intervention, Inner setting |
| 47 | Journal club | X |  |  |  | X | X |  |  | Learning | Education | Implementers, clinicians, outside consultants, intervention developers | Individual |
| 48 | Link to governance responsibility |  |  |  | X |  |  | X | X | Adaptation | environmental restructuring | Clinicians, administrators | Inner setting |
| 49 | Link to priorities | X |  |  |  | X | X | X | X | Commitment | Persuasion | Implementers, clinicians, patients, administrators, community stakeholders, outside consultants | Inner setting, Outer setting |
| 50 | Link to patient needs |  |  | X |  | X | X | X | X | Commitment | Persuasion | Patient, community stakeholder, clinician, implementer, administrator, intervention developers | Outer setting |
| 51 | Link to resources/ equipment |  |  | X |  |  | X | X | X | Adaptation | Environmental restructuring | Implementer, clinician, patient, intervention developer, administrator, outside consultant | Intervention |
| 52 | Publicize equipment | X |  |  |  | X | X |  |  | Marketing | Persuasion | Implementer, clinician, intervention developer, administrator, outside consultant | Inner setting |
| 53 | Mobile Roadshow |  |  | X |  | X | X | X | X | Marketing | Training | Implementers, clinicians, community stakeholders, outside consultants | Individual |
| 54 | Interprofessional discussion |  |  | X |  | X | X | X | X | Adaptation | persuasion | Implementers, clinicians, administrators | Inner setting |
| 55 | Professional roles |  |  | X |  | X | X | X | X | Adaptation | Environmental restructuring | Administrators, payers, outside consultants, implementers | Inner setting |
| 56 | Patient decision aid |  |  | X |  |  | X | X | X | Decision support | Enablement | Intervention developers, implementers, clinicians, patients, community stakeholders, | Outer setting |
| 57 | Patient reminder |  |  | X |  |  | X | X | X | Decision support | Enablement | Implementers, clinicians, patients, community stakeholders, intervention developers | Outer setting |
| 58 | Personalize message |  |  |  | X | X | X | X | X | Reinforcement | Persuasion | Implementers, patients, clinicians, administrators, outside stakeholders | Individuals |
| 59 | Pocket guide |  |  | X |  |  | X | X | X | Decision support | Restriction | Implementers, clinicians, patients, intervention developers, outside consultants | Inner setting |
| 60 | Resource material |  | X |  |  |  | X | X | X | Decision support | Education | Implementers, clinicians, patients, intervention developers, outside consultants, community stakeholders | Inner setting, individuals |
| 61 | Poster |  | X |  |  | X | X | X |  | Information | Education | Implementers, clinicians, outside consultants, intervention developers | Individuals |
| 62 | Flyer | X |  |  |  | X | X | X |  | Flyer | Enablement | Implementers, clinicians, outside consultants, intervention developers, administrators, community stakeholders | Individuals |
| 63 | Orientation |  | X |  |  |  | X |  | X | Information | Training | Implementers, clinicians, outside consultants | Individual |
| 64 | Staff meeting | X |  |  |  | X |  |  |  | Information | Education | Administrators, implementers, clinicians | Inner setting |
| 65 | Inservice |  | X |  |  | X | X |  |  | Information | Education | implementers, clinicians, outside consultant | Individuals |
| 66 | Newsletter | X |  |  |  | X |  |  |  | Information | Education | Administrators, implementers, clinicians, outside consultants, community stakeholders | Individuals |
| 67 | Learning communities |  |  | X |  | X | X | X |  | Learning | Enablement | Implementers, clinicians, administrators, outside consultants, intervention developers | Inner setting |
| 68 | Skill competence |  |  | X |  |  | X | X | X | Learning | Training | Implementers, clinicians, patients, outside consultants, intervention developer | Individuals |
| 69 | Clinician reminder |  |  | X |  |  | X | X | X | Decision support | Enablement | Implementers, clinicians, outside consultants, intervention developers | Individuals, inner setting |
| 70 | Organizational policy |  |  |  | X | X | X | X | X | Adaptation | Restriction | Implementers, administrators, clinicians | Inner setting |
| 71 | Role model |  |  | X |  | X | X | X | X | Adaptation | Modeling | Clinicians, patients, community stakeholders, administrators | Individuals |
| 72 | Rounding |  |  | X |  | X | X | X | X | Organizational infrastructure | Enablement | Administrators, clinicians, implementers | Outer setting, inner setting |
| 73 | Slogan and logo | X |  |  |  | X | X |  |  | Marketing | Persuasion | Implementers, outside consultants, intervention developers, clinicians, patients | Individuals |
| 74 | Sound bite | X |  |  |  | X | X |  |  | Marketing | Persuasion | Implementers, clinicians, patients, outside consultants, administrators, interventions developers | Individuals |
| 75 | Adapt for subgroup |  |  | X |  |  | X | X | X | Adaptation | Environmental restructuring | Implementers, clinicians, community stakeholders, outside consultants, intervention developers | Outer setting, intervention |
| 76 | Troubleshoot for individual |  |  | X |  |  | X | X | X | Adaptation | Enablement | Implementers, clinicians, patients | Individuals, intervention |
